# Minichromosome maintenance proteins in lung adenocarcinoma: Clinical significance and therapeutic targets

**DOI:** 10.1002/2211-5463.13681

**Published:** 2023-08-07

**Authors:** Kengo Tanigawa, Yuya Tomioka, Shunsuke Misono, Shunichi Asai, Naoko Kikkawa, Yoko Hagihara, Takayuki Suetsugu, Hiromasa Inoue, Keiko Mizuno, Naohiko Seki

**Affiliations:** ^1^ Department of Pulmonary Medicine, Graduate School of Medical and Dental Sciences Kagoshima University Japan; ^2^ Department of Functional Genomics Chiba University Graduate School of Medicine Japan

**Keywords:** lung adenocarcinoma, *MCM*, *miR‐139‐3p*, *miR‐2110*, *miR‐378a‐5p*, simurosertib

## Abstract

Lung cancer is the most common cause of cancer‐related death worldwide, accounting for 1.8 million deaths annually. Analysis of The Cancer Genome Atlas data showed that all members of the minichromosome maintenance (MCM) family (hexamers involved in DNA replication: *MCM2*‐*MCM7*) were upregulated in lung adenocarcinoma (LUAD) tissues. High expression of *MCM4* (*P* = 0.0032), *MCM5* (*P* = 0.0032), and *MCM7* (*P* = 0.0110) significantly predicted 5‐year survival rates in patients with LUAD. Simurosertib (TAK‐931) significantly suppressed the proliferation of LUAD cells by inhibiting cell division cycle 7‐mediated MCM2 phosphorylation. This finding suggested that MCM2 might be a therapeutic target for LUAD. Moreover, analysis of the epigenetic regulation of *MCM2* showed that *miR‐139‐3p*, *miR‐378a‐5p*, and *miR‐2110* modulated *MCM2* expression in LUAD cells. In patients with LUAD, understanding the role of these miRNAs may improve prognoses.

## Graphical abstract

Overexpression of MCM2‐MCM7 is closely associated with the molecular pathogenesis of LUAD. Control of these genes leads to control of lung cancer. We recognized the regulation of miRNAs as an epigenomic factor for MCMs overexpression. The *MCM2* were directly regulated by *miR‐139‐3p*, *miR‐378a‐5p*, and *miR‐2110* in LUAD cells.

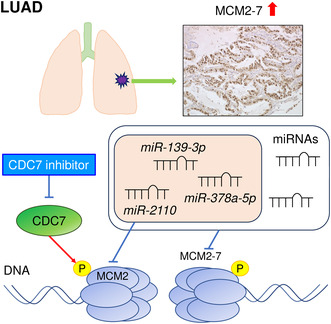

**Abbreviations**
CDC7: cell division cycle 7
DBF4: dumbbell former 4 protein
GAPDH: glyceraldehyde‐3‐phosphate dehydrogenase
GDC: Genomic Data Commons
GEO: Gene Expression Omnibus
GSEA: Gene Set Enrichment Analysis
IHC: immunohistochemistry
LUAD: lung adenocarcinoma
MCM: minichromosome maintenance
TCGA: The Cancer Genome Atlas

Lung cancer is the most common cause of cancer‐related death worldwide, accounting for 1.8 million deaths annually [1]. Lung cancer is classically divided into two types: small cell lung cancer (SCLC), which accounts for 15% of cases, and non‐small cell lung cancer (NSCLC), which accounts for 85% of cases [2, 3]. The main subtypes of NSCLC are lung adenocarcinoma (LUAD), lung squamous cell carcinoma, and large cell carcinoma. The most common histological subtype is LUAD, which is also the leading cause of cancer‐related deaths [4]. Recent genomic studies have revealed driver mutations that are characteristic of LUAD and molecularly targeted drugs have been developed based on these driver mutations [5, 6]. The survival rate in patients with lung cancer has increased owing to the development of effective treatment strategies, including molecularly targeted drugs and immune checkpoint inhibitors [5, 6, 7].

The prognosis of patients with LUAD is poor (the average 5‐year survival rate is below 20%), despite the availability of many treatment strategies [8]. Moreover, many patients with lung cancer are often diagnosed at an advanced stage, and metastasis to other organs occurs frequently [9, 10]. Unfortunately, effective treatment regimens for patients with metastatic lung cancer are limited [11]. Therefore, additional studies are needed to identify effective targeted molecular therapies for LUAD.

Minichromosome maintenance (MCM) family proteins are composed of six subunits (MCM2‐MCM7), which form a heterohexameric complex, and act as essential players in the initiation and elongation of eukaryotic DNA replication [12]. The MCM complex is also a replication gatekeeper that allows only one DNA replication per cell cycle, and knockdown of these genes induces cell cycle arrest and apoptosis in cancer cells [13]. Aberrant expression of MCM proteins has been reported in several types of cancers, for example, renal cell carcinoma, prostate cancer, and breast cancer [14, 15, 16].

Because overexpression of MCM proteins promotes the malignant transformation of cancer cells, anticancer drugs targeting MCMs are being studied [17, 18, 19]. Previous studies have shown that some antibiotics (e.g., heliquinomycin and ciprofloxacin) inhibit the helicase activity of MCMs and suppress the growth of cancer cells [18, 20]. Some statins, for example, lovastatin and atorvastatin, which inhibit HMG‐CoA reductase to lower blood cholesterol levels, have also been shown to suppress the expression of MCM2 and induce cell cycle arrest and apoptosis [19, 21]. Moreover, biologically active compounds isolated from natural products (e.g., breviscapine and cedrol) suppress the expression of MCMs and inhibit cancer cell proliferation [22, 23]. MCMs are therapeutic targets for a wide range of cancers, and control of these molecules may improve the prognosis of patients with cancers.

Numerous studies have shown that the noncoding RNAs in the human genome are key players in fine‐tuning gene expression [24, 25]. miRNAs are noncoding RNAs that control gene transcription levels by binding to specific sites in target RNAs [26, 27]. Notably, a single miRNA can control many RNA transcripts [25]. Therefore, aberrant expression of miRNAs can trigger the malignant transformation of human cells. Several studies have shown that aberrant expression of miRNAs occurs frequently in cancer cells and that their expression is closely involved in cancer cell progression, metastasis, and drug resistance [28, 29]. Previous studies have shown that *miR‐1296* directly regulates *MCM2* expression in prostate cancer cells [30]. Furthermore, *miR‐214‐3p* is downregulated in hepatocellular carcinoma cells, and this miRNA controls the expression of both *MCM5* and *MCM7* [31].

In this study, we investigated the clinical significance of all MCM family members (hexamers involved in DNA replication: *MCM2*‐*MCM7*) in LUAD cells. Analysis of The Cancer Genome Atlas (TCGA) data showed that all MCM family members (*MCM2*‐*MCM7*) were upregulated in LUAD tissues. Overexpression of all MCMs in LUAD clinical specimens was confirmed by immunostaining. Notably, high expression of *MCM4* (*P* = 0.0032), *MCM5* (*P* = 0.0032), and *MCM7* (*P* = 0.0110) significantly predicted 5‐year survival rates in patients with LUAD. Functional assays showed that MCM2 was a promising therapeutic target in LUAD cells. Simurosertib significantly suppressed the proliferation of LUAD cells by inhibiting MCM2 phosphorylation.

Finally, we investigated the epigenetic regulation of *MCM2* and found that *miR‐139‐3p*, *miR‐378a‐5p*, and *miR‐2110* controlled *MCM2* expression in LUAD cells. Through their functions as DNA helicases, MCM family members were found to be essential for the replication of genomic DNA and were closely involved in the molecular pathogenesis of LUAD. In particular, MCM2 may be a therapeutic target in patients with LUAD, and control of this molecule may improve prognoses in patients with LUAD.

## Materials and methods

### *In silico* analysis of gene expression levels and prognosis

The clinical significance of genes in LUAD was evaluated using TCGA datasets (https://www.cancer.gov/tcga). Data on gene expression levels were obtained from FIREBROWSE (http://firebrowse.org/) and the Genomic Data Commons (GDC) Data Portal (https://portal.gdc.cancer.gov/). The overall survival data were obtained from cBioPortal (https://www.cbioportal.org/) [32] and OncoLnc (http://www.oncolnc.org/) (data downloaded on March 26, 2021) [33].

### Cell lines and cell culture

Two NSCLC cell lines, A549 and H1299, were used in this study (American Type Culture Collection, Manassas, VA, USA). Both cell lines were grown in RPMI‐1640 medium (FUJIFILM Wako Pure Chemical Corporation, Osaka, Japan) supplemented with 10% fetal bovine serum. Both cell lines were cultured in an incubator at 37 °C in an atmosphere containing 5% CO_2_. Cells were cultured as described previously [34]. Confirmation of negativity for *Mycoplasma* infection in A549 and H1299 cells was carried out using a LookOut Mycoplasma PCR Detection Kit (catalog no.: MP0035; Sigma‐Aldrich, MO, USA) and JumpStart Taq DNA Polymerase (catalog no.: D9307; Sigma‐Aldrich) according to the manufacturer's protocol.

### Transfection with small interfering RNA (siRNA) and miRNA

Opti‐MEM (Gibco, Carlsbad, CA, USA) and Lipofectamine RNAiMax Transfection Reagent (Invitrogen, Carlsbad, CA, USA) were used to transfect siRNA and miRNA into cell lines. The procedure for siRNA and miRNA transfection was described previously [34, 35, 36]. The siRNAs and miRNAs used in this study are listed in Table S1.

### RNA extraction and reverse transcription‐quantitative PCR

Total RNA from NSCLC cell lines was extracted using Isogen II (NIPPON GENE Co., Ltd., Tokyo, Japan). A NanoDrop 2000c spectrophotometer (Thermo Fisher Scientific Inc., Waltham, MA, USA) was used to check the quantity and quality of total RNA. cDNA was synthesized using a PrimeScript RT Master Mix (catalog no.: RR036A; Takara Bio Inc., Shiga, Japan). TaqMan Real‐Time PCR Assays were conducted to analyze gene expression, as described previously [34, 35, 36]. Reverse transcription‐quantitative PCR (RT‐qPCR) was performed using a StepOnePlus Real‐Time PCR System (Applied Biosystems, Foster City, CA, USA). The reagents used in this study are listed in Table S1.

### Western blotting

Non‐small cell lung cancer cells were lysed with RIPA Lysis Buffer (catalog no.: sc‐24948; Santa Cruz Biotechnology Inc., Dallas, TX, USA). When we extract phosphorylated proteins, PhosSTOP (catalog no.: 04906845001; Roche, Mannheim, Germany) was added to the buffer system. A Pierce BCA Protein Assay Kit (Thermo Fisher Scientific, Rockford, IL, USA) was used to assay protein concentrations. SuperSep Ace (7.5%, 13‐well; FUJIFILM Wako Pure Chemical Corporation) was used for sodium dodecyl sulfate‐polyacrylamide gel electrophoresis. Precision Plus Protein Dual WesternC Standards (catalog no.: 161‐0376; Bio‐Rad Laboratories, Inc., Herclules, CA, USA) were used as the standard. Proteins were transferred on polyvinylidene fluoride membranes (catalog no.: PPVH00010; Merck KGaA, Darmstadt, Germany). The membranes were blocked with 3% bovine serum albumin (catalog no.: 01863‐77; Nacalai Tesque, Inc., Kyoto, Japan) in TBST. Precision Protein Strep Tactin‐HRP Conjugate (catalog no.: 1610380; Bio‐Rad Laboratories, Inc.) was used to detect the standard. The signal was developed using Amersham ECL Prime Western Blotting Detection Reagent (Cytiva, Marlborough, MA, USA). Chemiluminescence was performed using FluorChem FC2 (Cell Biosciences, Santa Clara, CA, USA) to visualize western blotting. The reagents used in this study are listed in Table S1.

### Cell proliferation and cell cycle assays

Cell proliferation was evaluated using a Cell Proliferation Kit (XTT based; Biological Industries, Beit‐Haemek, Israel). BD Cycletest Plus DNA Reagent Kit (BD Biosciences, Franklin Lakes, NJ, USA) was used for the evaluation of the cell cycle distribution on a BD FACSCelesta Flow Cytometer (BD Biosciences). The data were analyzed using FlowJo software (TreeStar, Ashland, OR, USA). The procedures for cell proliferation and cell cycle analyses were described previously [34, 35, 36].

### Immunohistochemical staining

Tissue microarray slides (catalog no.: LC811a; US Biomax, Inc., Derwood, MD, USA) were used for immunohistochemical staining. Blocking, the primary antibody reaction, secondary antibody reaction, and binding of avidin to the biotin complex were conducted using a VECTASTAIN Universal Elite ABC Kit (Vector Laboratories, Burlingame, CA, USA) according to the manufacturer's protocol. Dako Real antibody diluent (Agilent, Santa Clara, CA, USA) was used to dilute the primary antibody. The chromogenic reaction was developed using a Dako REAL EnVision Detection System Peroxidase/DAB+, Rabbit/Mouse (Agilent). The primary antibody used in this study is described in Table S1. The characteristics of patients from whom the tissue samples were derived in this tissue array are described in Table S2. The intensity and area of staining were evaluated based on previous reports [37], and immunohistochemical scores were calculated.

### Cytotoxicity assay

For cytotoxicity assays, 100 μL of cell suspension (approximately 2 × 10^3^ cells·mL^−1^) was seeded in each well of a 96‐well plate, and cells were allowed to attach overnight. The next day, culture media were discarded, and the cells were treated with different concentrations of TAK‐931 (catalog no.: HY‐100888/CS‐0020550; MedChemExpress, NJ, USA) ranging from 1 to 1000 nm. The plates were incubated for 5 days, and media containing TAK‐931 were replaced every other day. Cells treated with 0.1% DMSO served as a negative control. To investigate cell toxicity, XTT assays were performed using a Cell Proliferation Kit (Biological Industries) according to the manufacturer's protocol. The signals were measured using a microplate reader (Bio‐Rad Laboratories, Inc.).

### Colony formation assay

For colony formation assays, 1 mL medium containing 300 cells was added to each well of a 12‐well plate, and the cells were treated with different concentrations of TAK‐931 the next day. TAK‐931 was replaced every other day, and the cells were incubated for 10 consecutive days. Cells treated with 0.1% DMSO were used as a control. At the end of the incubation, the colonies were washed with phosphate‐buffered saline and subsequently fixed using 4% paraformaldehyde for 15 min at room temperature. After fixation, the colonies were dyed with 0.5% crystal violet (FUJIFILM Wako Pure Chemical Corporation, Osaka, Japan) for 25 min. The wells were washed with water, and the plates were air‐dried.

### Identification of putative miRNAs binding to *MCM2*‐*MCM7* and analysis of miRNA expression

Data for putative miRNAs binding to *MCM2‐MCM7* were obtained from the targetscanhuman database (release 8.0) (https://www.targetscan.org/vert_80/) [38]. Data for miRNA expression levels were obtained from FIREBROWSE (http://firebrowse.org/) and the GDC Data Portal (https://portal.gdc.cancer.gov/) (data downloaded on August 4, 2022).

### The construction of plasmid and dual‐luciferase reporter assays

The wild‐type sequences containing miRNAs binding sites in the 3′‐untranslated regions (UTRs) of *MCM2* and the deletion‐type sequences lacking them were cloned into the psiCHECK‐2 vector (C8021; Promega, Madison, WI, USA). The procedures for transfection and dual‐luciferase reporter assays were provided in previous studies [34, 35].

### MCM family alteration analysis based GSEA analysis

The data were obtained from cBioPortal (http://cbioportal.org, accessed on May 19, 2023 [32]). We executed queries to specify *MCM2‐MCM7* mRNA expression changes (*Z*‐score ≥0) and analyzed survival. Based on mRNA expression levels compared between altered and non‐altered groups, the ranked gene list for Gene Set Enrichment Analysis (GSEA) was obtained and uploaded into WEB‐based GEne SeT AnaLysis Toolkit, ‘WebGestalt’ (http://www.webgestalt.org), accessed on May, 19 2023 [39]. We applied ‘Wikipathway cancer’ dataset for GSEA (https://www.wikipathways.org/index.php/WikiPathways, accessed on May 19, 2023 [40]).

### Statistical analysis

Statistical analyses were performed using graphpad prism 8 (GraphPad Software, La Jolla, CA, USA) and jmp pro 14 (SAS Institute Inc., Cary, NC, USA). Differences between the two groups were analyzed using Student's *t*‐tests or Mann–Whitney *U*‐tests. Multiple group comparisons were performed using one‐way ANOVA and Tukey tests for *post hoc* analyses. Survival rates were analyzed using Kaplan–Meier survival curves and log‐rank tests.

## Results

### Expression and clinical significance of *MCM2*‐*MCM7* in patients with LUAD by TCGA analysis

First, we analyzed the Gene Expression Omnibus (GEO) database (GEO accession no: GSE19188) to identify genes exhibiting oncogenic effects in NSCLC. The upregulated genes in GSE19188 were narrowed down based on the criteria of log_2_ (fold change) greater than 1 and *P*‐value less than 0.05; using these parameters, 613 genes were identified. These candidate genes were classified according to the Kyoto Encyclopedia of Genes and Genomes pathways by GeneCodis 4 database (https://genecodis.genyo.es/) (data downloaded on March 17, 2021) [41]. The top 10 annotations based on relative enrichment score were mucin type O‐glycan biosynthesis, DNA replication, glycosaminoglycan biosynthesis—heparan sulfate/heparin, RNA degradation, homologous recombination, protein digestion and absorption, Fanconi anemia pathway, cell cycle, ether lipid metabolism, and glycosphingolipid biosynthesis—lacto and neolacto series (Table 1). In our previous reports, we found that cell cycle and DNA replication genes regulated by miRNAs affected lung cancer progression. Twenty‐four and eight genes were classified as cell cycle‐ and DNA replication‐related genes, respectively (Tables 2 and 3). *MCM2*, *MCM4*, *MCM6*, and *MCM7* were common in these two annotations.

**Table 1:** Enriched annotations of the upregulated genes identified by GSE19188.

Annotation	Annotation ID	Number of genes	Relative enrichment score	*P*‐value	Adjusted *P*‐value	Genes
Mucin‐type O‐glycan biosynthesis	hsa00512	3	50.31	< 0.001	< 0.001	*GCNT3, GALNT14, GALNT7*
DNA replication	hsa03030	8	47.36	< 0.001	< 0.001	*RNASEH2A, RFC4, POLE2, MCM7, MCM6, MCM4, MCM2, FEN1*
Glycosaminoglycan biosynthesis—heparan sulfate/heparin	hsa00534	2	40.25	< 0.001	0.003	*HS3ST1, HS6ST2*
RNA degradation	hsa03018	4	33.54	< 0.001	< 0.001	*PFKP, HSPD1, ENO1, PABPC1L*
Homologous recombination	hsa03440	5	29.60	< 0.001	< 0.001	*RAD54B, BLM, BARD1, RAD51, BRIP1*
Protein digestion and absorption	hsa04974	9	29.22	< 0.001	< 0.001	*KCNN4, COL12A1, COL11A1, COL10A1, COL5A2, COL5A1, COL3A1, COL1A2, COL1A1*
Fanconi anemia pathway	hsa03460	6	28.75	< 0.001	< 0.001	*BLM, RAD51, RMI2, BRIP1, FANCI, UBE2T*
Cell cycle	hsa04110	24	27.44	< 0.001	< 0.001	*CHEK1, CDKN2A, CDC25C, CDC20, CDC6, CDK1, ESPL1, CCNE1, CCNB1, CCNA2, PTTG1, CCNE2, CCNB2, CDC45, TTK, BUB1B, BUB1, TFDP2, PLK1, MCM7, MCM6, MCM4, MCM2, MAD2L1*
Ether lipid metabolism	hsa00565	5	26.48	< 0.001	< 0.001	*PLPP2, UGT8, PLA2G4A, PAFAH1B3, EPT1*
Glycosphingolipid biosynthesis—lacto and neolacto series	hsa00601	3	25.16	< 0.001	0.001	*B3GNT3, FUT3, FUT2*

**Table 2:** Upregulated genes associated with cell cycle.

Entrez GeneID	Gene symbol	Gene name	Log_2_ fold change GSE19188	*P*‐value GSE19188
991	*CDC20*	Cell division cycle 20	2.73	< 0.001
891	*CCNB1*	Cyclin B1	2.64	< 0.001
7272	*TTK*	TTK protein kinase	2.49	< 0.001
9133	*CCNB2*	Cyclin B2	2.36	< 0.001
4085	*MAD2L1*	Mitotic arrest deficient 2 like 1	2.30	< 0.001
701	*BUB1B*	BUB1 mitotic checkpoint serine/threonine kinase B	2.25	< 0.001
983	*CDK1*	Cyclin‐dependent kinase 1	2.10	< 0.001
699	*BUB1*	BUB1 mitotic checkpoint serine/threonine kinase	2.06	< 0.001
890	*CCNA2*	Cyclin A2	1.92	< 0.001
4173	*MCM4*	Minichromosome maintenance complex component 4	1.78	< 0.001
1111	*CHEK1*	checkpoint kinase 1	1.75	< 0.001
4171	*MCM2*	Minichromosome maintenance complex component 2	1.71	< 0.001
898	*CCNE1*	Cyclin E1	1.71	< 0.001
1029	*CDKN2A*	Cyclin‐dependent kinase inhibitor 2A	1.67	< 0.001
990	*CDC6*	Cell division cycle 6	1.63	< 0.001
9232	*PTTG1*	PTTG1 regulator of sister chromatid separation, securin	1.59	< 0.001
9134	*CCNE2*	Cyclin E2	1.57	< 0.001
8318	*CDC45*	Cell division cycle 45	1.47	< 0.001
4176	*MCM7*	Minichromosome maintenance complex component 7	1.24	< 0.001
995	*CDC25C*	Cell division cycle 25C	1.11	< 0.001
5347	*PLK1*	Polo‐like kinase 1	1.09	< 0.001
4175	*MCM6*	Minichromosome maintenance complex component 6	1.09	< 0.001
9700	*ESPL1*	Extra spindle pole bodies like 1, separase	1.06	< 0.001
7029	*TFDP2*	Transcription factor Dp‐2	1.02	< 0.001

**Table 3:** Upregulated genes associated with DNA replication.

Entrez GeneID	Gene symbol	Gene name	Log_2_ fold change GSE19188	*P*‐value GSE19188
4173	*MCM4*	Minichromosome maintenance complex component 4	1.78	< 0.001
4171	*MCM2*	Minichromosome maintenance complex component 2	1.71	< 0.001
5984	*RFC4*	Replication factor C subunit 4	1.59	< 0.001
10,535	*RNASEH2A*	Ribonuclease H2 subunit A	1.28	< 0.001
2237	*FEN1*	Flap structure‐specific endonuclease 1	1.28	< 0.001
5427	*POLE2*	DNA polymerase epsilon 2, accessory subunit	1.24	< 0.001
4176	*MCM7*	Minichromosome maintenance complex component 7	1.24	< 0.001
4175	*MCM6*	Minichromosome maintenance complex component 6	1.09	< 0.001

**Fig. 1:**
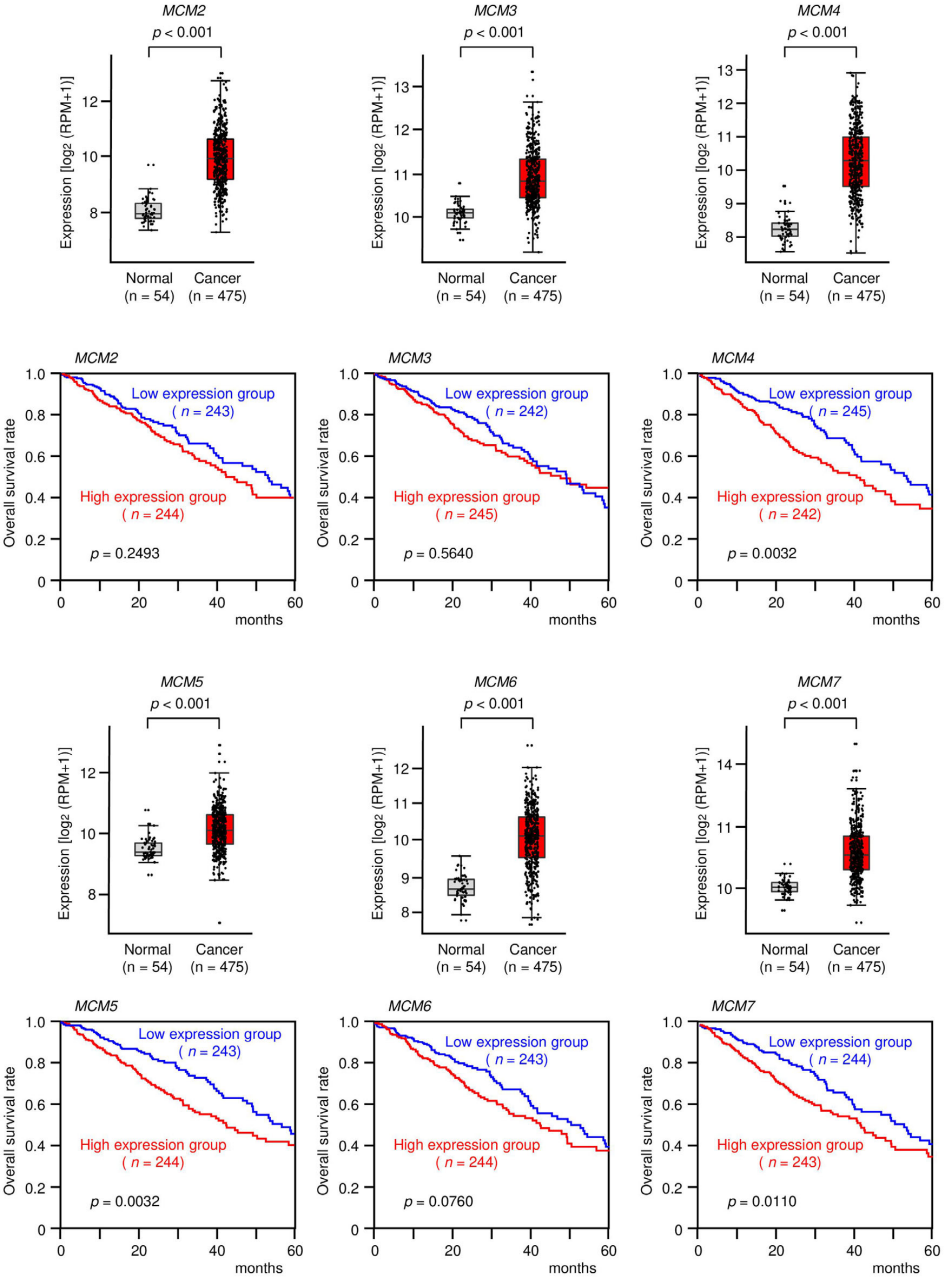
Expression levels of *MCM2*, *MCM3*, *MCM4*, *MCM5*, *MCM6*, and *MCM7* and association with overall survival in patients with LUAD. The expression levels of six genes (*MCM2*, *MCM3*, *MCM4*, *MCM5*, *MCM6*, and *MCM7*) in normal and cancer tissues were evaluated using data from TCGA database. In total, 475 LUAD tissues and 54 normal lung tissues were analyzed. The lower and upper hinges represent the 25th and 75th percentiles, respectively. The lower/upper whiskers represent the lowest/largest points inside the range defined by the first/third quartiles ± 1.5 times the IQR, respectively. Dots represent individual samples. Student's *t*‐test. Kaplan–Meier 5‐year overall survival curves were created from TCGA data. Patients were divided into high‐ and low‐expression groups according to miRNA expression (based on median expression). The red line shows the high‐expression group, and the blue line shows the low‐expression group. Log‐rank test. RPM, reads per million.

MCM2‐MCM7 form a hexamer and act as a DNA helicase essential for genomic DNA replication. Notably, *MCM2‐MCM7* were significantly upregulated in LUAD tissues compared with normal tissues, and the 5‐year overall survival rates tended to be poor in patients with high *MCM2‐MCM7* expression (Fig. 1). In this study, we focused on *MCM2‐MCM7*.

### Overexpression of MCM2‐MCM7 in LUAD clinical specimens

**Fig. 2:**
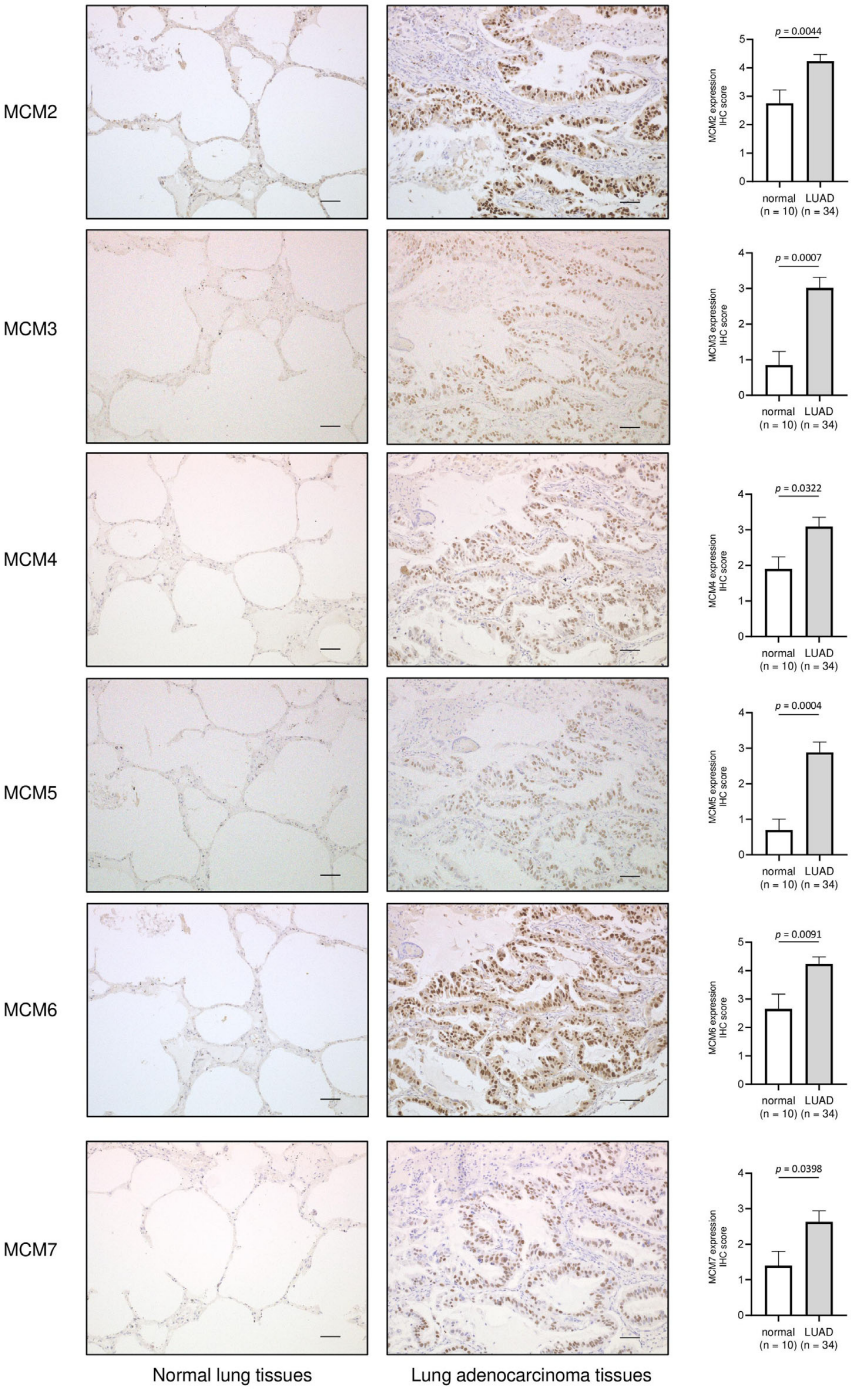
MCM2, MCM3, MCM4, MCM5, MCM6, and MCM7 expression levels in LUAD clinical specimens. Expression levels of MCM2, MCM3, MCM4, MCM5, MCM6, and MCM7 in LUAD and normal lung tissues were evaluated by IHC using a tissue microarray. Representative LUAD tissues (patient no. 9) and normal lung tissues (patient no. 75) are shown. The data are means and standard errors of the means. Mann–Whitney *U*‐tests. Scale bars = 50 μm.

We performed immunohistochemistry (IHC) to investigate MCM2–MCM7 protein expression in LUAD. The nuclear expression of MCM2‐MCM7 was high in LUAD but low in normal lung tissues. MCM2‐MCM7 were significantly overexpressed in LUAD compared with normal lung tissues (Fig. 2). The characteristics of patients are described in Table S2.

### Search for miRNAs that negatively regulate *MCMs* (*MCM2*–*MCM7*) expression in LUAD cells

**Fig. 3:**
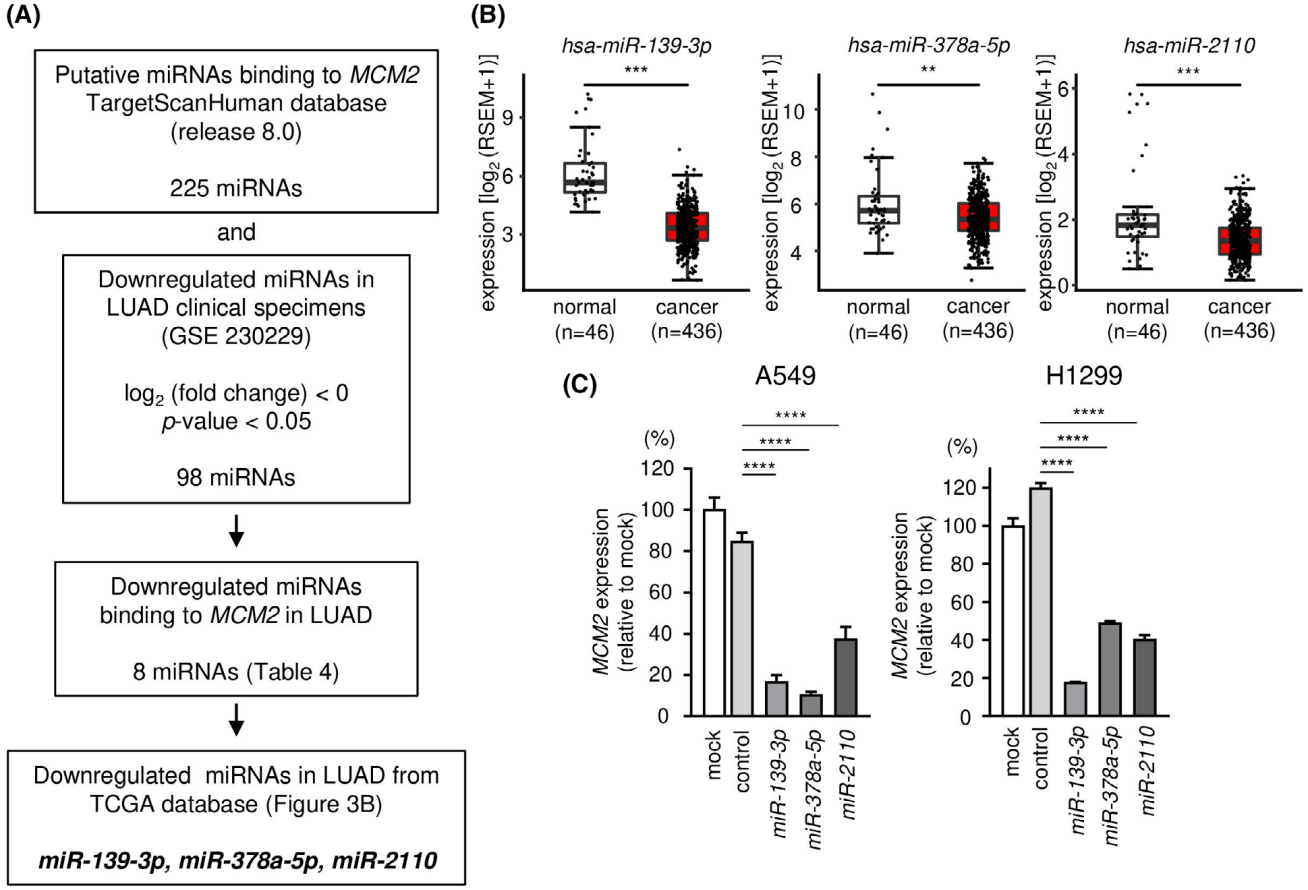
Selection of miRNAs regulating *MCM2* in LUAD cells. (A) This shows a flowchart for the strategy to identify the candidate of miRNAs regulating *MCM2* in LUAD cells. (B) The expression levels of *miR‐139‐3p*, *miR‐378a‐5p*, and *miR‐2110* were evaluated in a LUAD dataset from TCGA. A total of 436 LUAD tissues and 46 normal lung tissues were analyzed. The lower and upper hinges represent the 25th and 75th percentiles, respectively. The lower/upper whiskers represent the lowest/largest points inside the range defined by first/third quartile ± 1.5 times IQR, respectively. Dots mean each sample. Student's *t*‐test. (C) mRNA expressions of *MCM2* were evaluated by RT‐qPCR. Internal control was the expression of *GAPDH*. The time for transfection is 72 h. RT‐qPCR was measured in triplicate. The data are mean and SD. One‐way ANOVA and Tukey tests for *post hoc* analysis. **P < 0.01, ***P < 0.001, ****P < 0.001. RSEM, RNA‐seq by Expectation‐Maximization.

We hypothesized that one of the reasons for the overexpression of *MCMs* in LUAD cells is that some miRNAs expression is repressed. We recently created the RNA‐sequencing‐based miRNA expression signature using LUAD clinical specimens (GEO accession no: GSE230229). Based on this signature, we selected miRNAs whose expression is repressed in LUAD tissues. Next, we integrated these downregulated miRNAs with TargetScanHuman database (release 8.0) (https://www.targetscan.org/vert_80/), and selected miRNAs that may negatively regulate *MCMs* (*MCM2*–*MCM7*) in LUAD cells. Our strategy for miRNA selection is shown in Fig. 3A and Fig. S1. Candidate miRNAs that negatively regulated *MCM2*–*MCM7* are listed in Table 4 and Tables [Link], [Link].

**Table 4:** Candidate tumor‐suppressive miRNAs binding to *MCM2*. FDR, false discovery rate.

miRNA	miRBase accession No.	Log_2_ fold change GSE230229	Normalized read count GSE230229		FDR GSE230229	*P*‐value GSE230229
			LUAD tissues	Normal lung tissues		
*hsa‐miR‐603*	MIMAT0003271	−2.90	0.00	2.90	< 0.001	< 0.001
*hsa‐miR‐373‐3p*	MIMAT0000726	−2.09	0.00	2.09	0.152	0.038
*hsa‐miR‐139‐3p*	MIMAT0004552	−1.83	5.04	6.87	0.047	0.009
*hsa‐miR‐4732‐3p*	MIMAT0019856	−1.72	3.70	5.42	0.014	0.002
*hsa‐miR‐4516*	MIMAT0019053	−1.38	3.56	4.94	0.164	0.042
*hsa‐miR‐378a‐5p*	MIMAT0000731	−0.80	8.58	9.38	0.010	0.002
*hsa‐miR‐2110*	MIMAT0010133	−0.69	6.91	7.59	0.141	0.034
*hsa‐miR‐6513‐3p*	MIMAT0025483	−0.58	5.51	6.10	0.186	0.049

To verify the effectiveness of our list, we performed further analysis using *MCM2* as a model case. Our strategy identified a candidate of eight miRNAs that negatively controlled the expression of *MCM2* in LUAD cells (Fig. 3A and Table 4). Among these miRNAs, three miRNAs (*miR‐139‐3p*, *miR‐378a‐5p*, and *miR‐2110*) were significantly downregulated in LUAD specimens based on TCGA‐LUAD database analysis (Fig. 3B). *MCM2* mRNA expressions were significantly suppressed by ectopic expression of these miRNAs (*miR‐139‐3p*, *miR‐378a‐5p*, and *miR‐2110*) in LUAD cells (Fig. 3C).

**Fig. 4:**
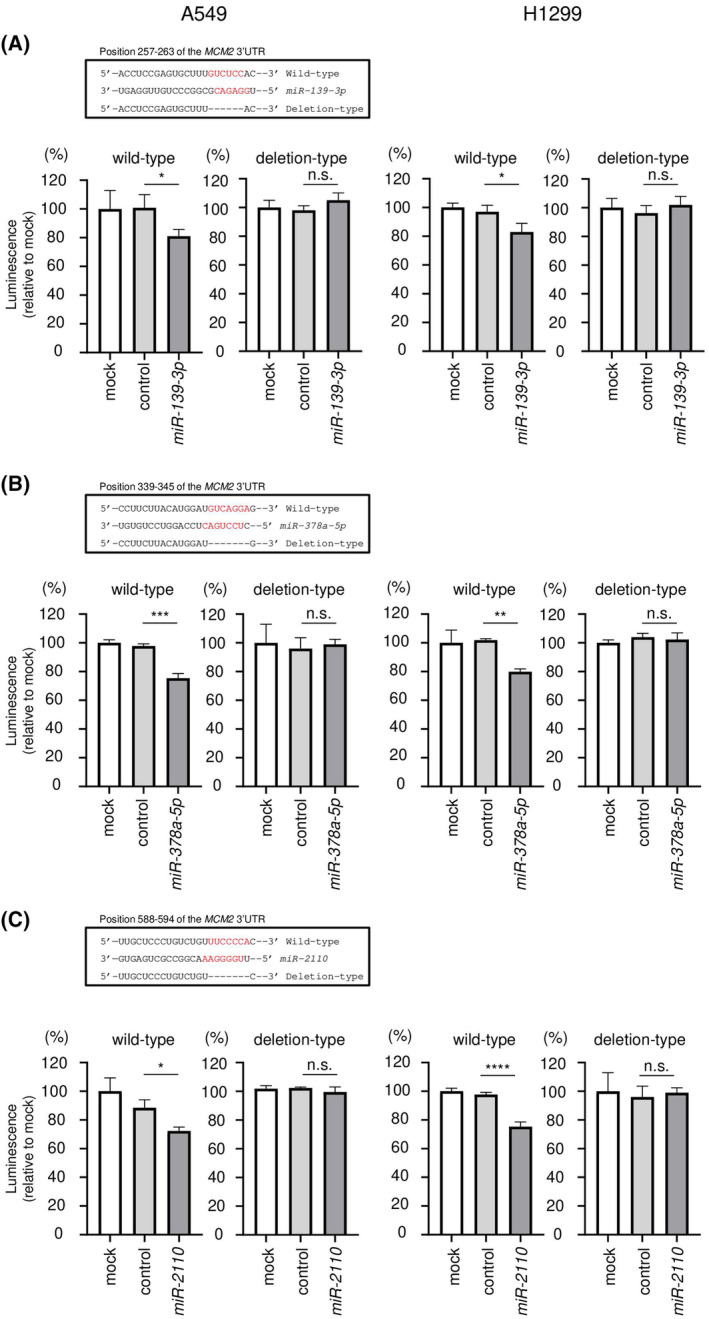
Direct regulation of *MCM2* expression by *miR‐139‐3p*, *miR‐378a‐5p*, and *miR‐2110* in LUAD cells. This figure shows dual‐luciferase reporter assay results following transfection with (A) *miR‐139‐3p*, (B) *miR‐378a‐5p*, and (C) *miR‐2110*. The upper panels are putative *miR‐139‐3p*, *miR‐378a‐5p*, and *miR‐2110* binding sites predicted within the 3′‐UTR of *MCM2* by TargetScanHuman database analysis. After 24 h co‐transfection of miRNA and wild‐type or deletion‐type psiCHECK‐2 vector, dual‐luciferase reporter assay was conducted (lower panel). Wild‐type vector contains the miRNA binding site of *MCM2* 3′‐UTR sequence, and deletion‐type one does not. Normalized data were calculated as the *Renilla*/firefly luciferase activity ratio. Luminescence activity was measured in triplicate. The data are mean and SD. One‐way ANOVA and Tukey tests for *post hoc* analysis. *P < 0.05, **P < 0.01, ***P < 0.001, ****P < 0.0001, n.s., not significant. RSEM, RNA‐seq by Expectation‐Maximization.

Furthermore, we examined whether these miRNAs (*miR‐139‐3p*, *miR‐378a‐5p*, and *miR‐2110*) directly bind to 3′UTR of *MCM2* by dual‐luciferase reporter assays. TargetScanHuman database shows the putative *miR‐139‐3p*‐binding site in the 3′‐UTR of *MCM2* (Fig. 4A upper). Luminescence intensity was significantly reduced following co‐transfected with *miR‐139‐3p* and a wild‐type vector (containing the *miR‐139‐3p* binding site in the 3′UTR of *MCM2*) (Fig. 4A lower). By contrast, no change in luminescence intensity following co‐transfected with *miR‐139‐3p* and a deletion‐type vector (without the *miR‐139‐3p* binding site in the 3′UTR of *MCM2*) (Fig. 4A lower). These results indicated that *miR‐139‐3p* directly bound to 3′UTR of *MCM2* and controlled *MCM2* expression in LUAD cells. Similar analyses were performed for the other miRNAs (*miR‐378a‐5p* and *miR‐2110*). The results revealed that two miRNAs also directly bound to the 3′UTR of *MCM2* and negatively regulate its expression in LUAD cells (Fig. 4B,C).

### Alteration of mRNA expression of *MCMs* (*MCM2*–*MCM7*) in LUAD patients based on TCGA‐LUAD analysis

We investigated the clinical impact of alteration of *MCMs* (*MCM2*–*MCM7*) expression in patients with LUAD using TCGA‐LUAD cohort dataset.

**Fig. 5:**
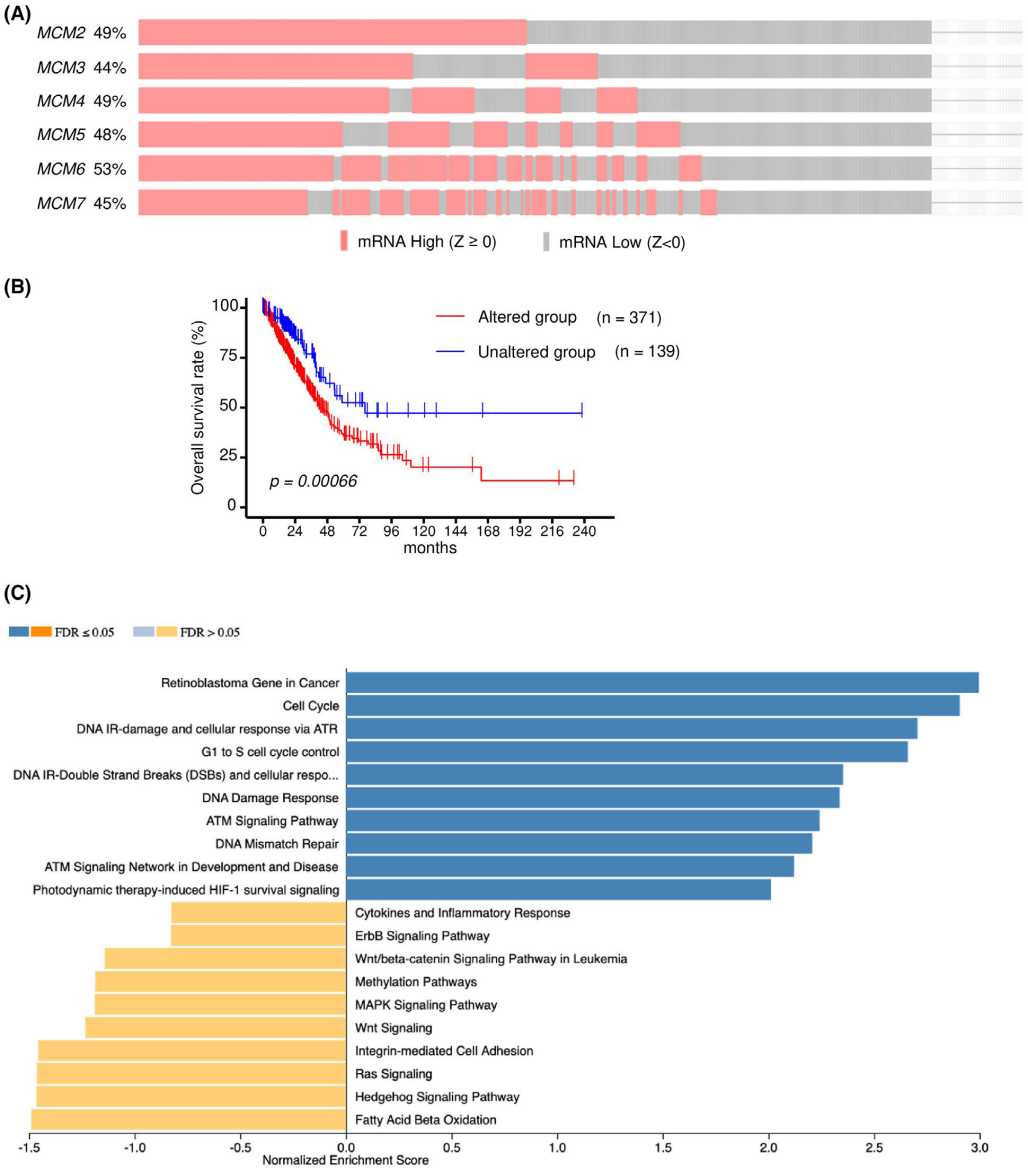
*MCM* family mRNA expression alternations in LUAD clinical specimens from TCGA‐LUAD analyses. (A) Oncoprint of TCGA‐LUAD on cBioPortal filtered by the mRNA expression (*Z*‐score ≥0) from the query for genes. (B) Kaplan–Meier curves of overall survivals between altered and unaltered groups. (C) Bar chart and enrichment plots of GSEA of alteration of MCM family. FDR, false discovery rate.

In 586 LUAD clinical samples, a large number of LUAD cohort data showed high mRNA expression for *MCM2* at 49%, *MCM3* at 44%, *MCM4* at 49%, *MCM5* at 48%, *MCM6* at 53%, and *MCM7* at 45% (*Z*‐score >0) (Fig. 5A). Interestingly, we found that there were many cases in which the expression of all 6 *MCM* genes was simultaneously increased (Fig. 5A).

Patients with LUAD who had high expression in at least one of the *MCM* genes (*MCM2*–*MCM7*) showed a worse survival outcome compared with patients with no *MCM* gene alteration (Fig. 5B). Gene set enrichment analysis using TCGA‐LUAD dataset revealed that ‘Retinoblastoma Gene in Cancer’, ‘Cell Cycle’, ‘DNA IR‐damage and cellular response via ATR’, and ‘G1 to S cell cycle control’ were identified as enrichment pathways in high expression of *MCM* genes group (Fig. 5C).

### Effects of *MCM2*‐*MCM7* knockdown on cell proliferation and the cell cycle

**Fig. 6:**
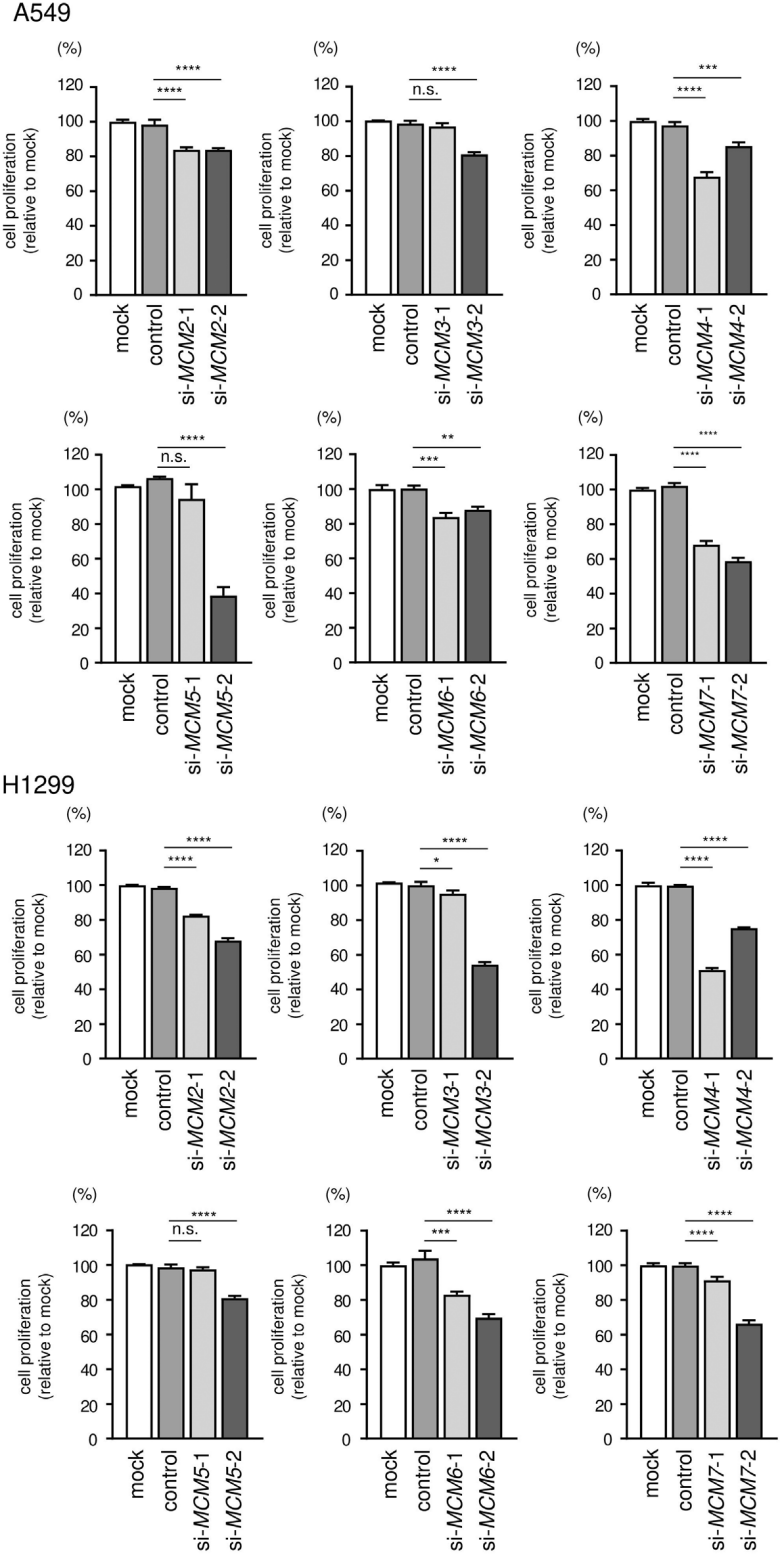
Cell proliferation assays after knockdown of *MCM2*, *MCM3*, *MCM4*, *MCM5*, *MCM6*, and *MCM7*. Cell proliferation after knockdown of *MCM2*, *MCM3*, *MCM4*, *MCM5*, *MCM6*, and *MCM7* was assessed using XTT assays. The transfection time was 96 h. Cell proliferation was measured in triplicate. The data are means and standard deviations. One‐way ANOVA and Tukey tests were used for *post hoc* analysis. *P < 0.05; **P < 0.01; ***P < 0.001; ****P < 0.0001. n.s., not significant.

**Fig. 7:**
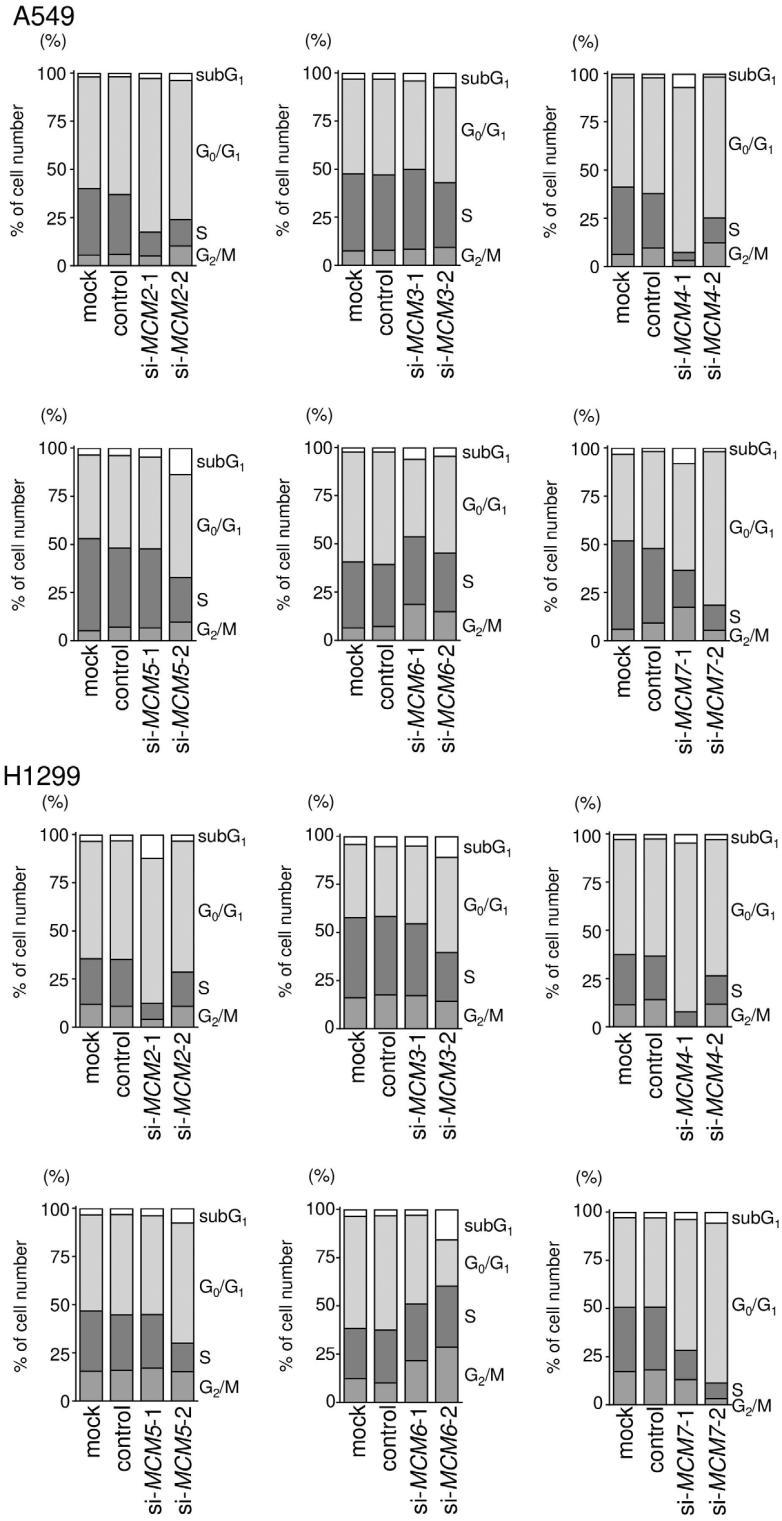
Cell cycle assays after knockdown of *MCM2*, *MCM3*, *MCM4*, *MCM5*, *MCM6*, and *MCM7*. Cell cycle distribution was tested by flow cytometry. The samples were evaluated 72 h after transfection with siRNAs targeting *MCM2*, *MCM3*, *MCM4*, *MCM5*, *MCM6*, and *MCM7*. Cell cycle distribution was measured in triplicate. Data in the stacked bar graphs are means.

Next, we investigated the effects of *MCM2‐MCM7* knockdown to evaluate the functions of *MCM2‐MCM7* in A549 and H1299 cells. First, we confirmed the knockdown efficiencies of *MCM2‐MCM7* siRNAs using RT‐qPCR and western blotting. Transfection with two siRNAs reduced the mRNA and protein expression of MCM2‐MCM7 in A549 and H1299 cells (Figs S2 and S3). Next, we conducted cell proliferation assays. Most siRNAs targeting *MCM2‐MCM7* suppressed cell proliferation (Fig. 6). Lastly, cell cycle assays were performed after transfection with siRNAs targeting *MCM2‐MCM7*. siRNAs targeting *MCM2‐MCM5* and *MCM7* led to G_0_/G_1_ arrest, and those targeting *MCM6* induced G_2_/M arrest (Fig. 7; Figs S4 and S5). These results suggested that knockdown of *MCM2‐MCM7* inhibited cell proliferation and cell cycle progression in A549 and H1299 cells.

### Effects of the CDC7 inhibitor TAK‐931 on A549 and H1299 cells

**Fig. 8:**
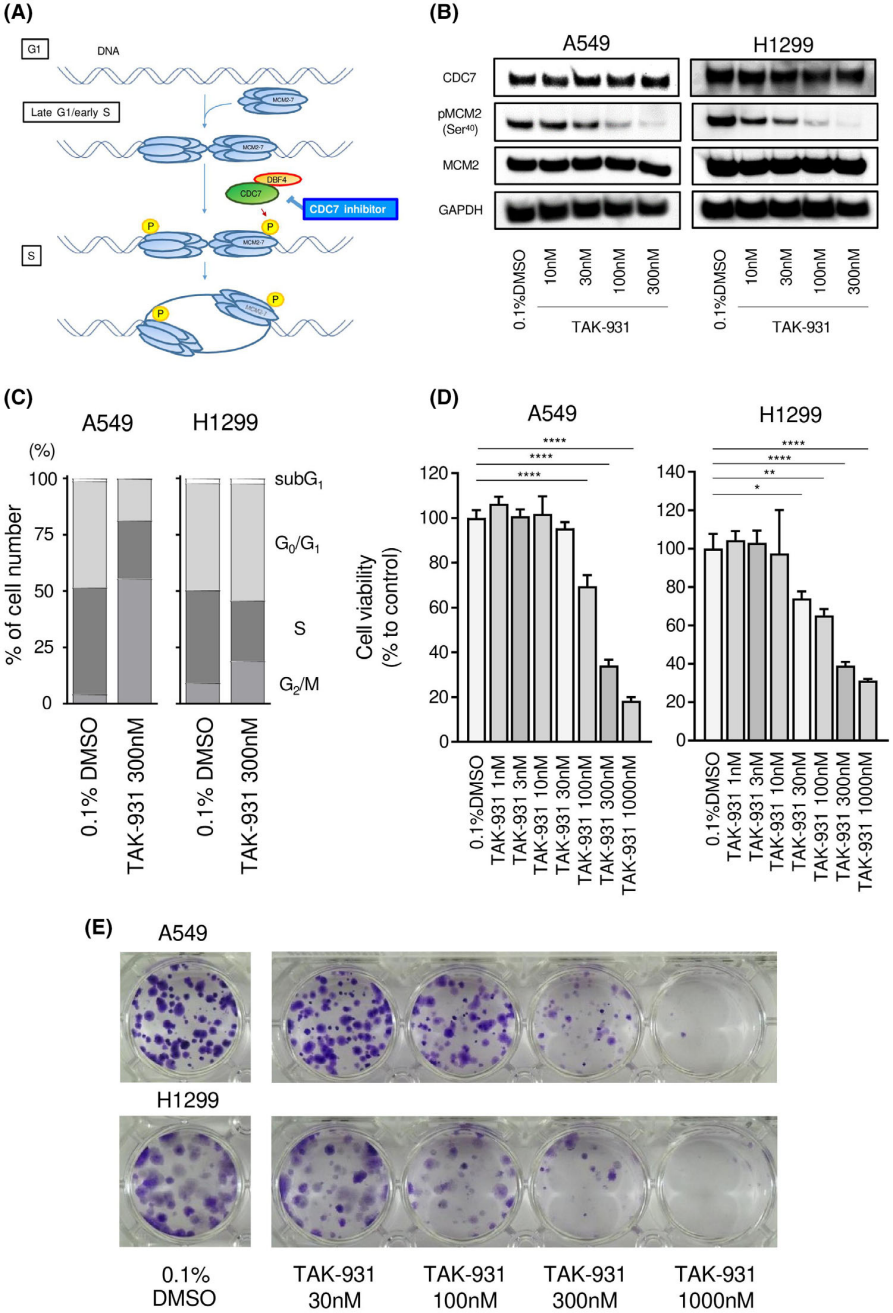
Effects of CDC7 inhibition on the phosphorylation of MCM2 and tumor progression in NSCLC. (A) The scheme shows the function of the CDC7 inhibitor. (B) Protein levels of CDC7, phospho‐MCM2, and MCM2 were evaluated by western blot analysis. Glyceraldehyde‐3‐phosphate dehydrogenase was used as a loading control. (C) Cell cycle distribution after treatment with TAK‐931 was tested by flow cytometry. The samples were evaluated 24 h after TAK‐931 administration. Cell cycle distribution was measured in triplicate. (D) Cytotoxicity was evaluated using XTT assays. The treatment duration was 5 days. Cytotoxicity assays were performed in triplicate. Data are means and standard deviations. One‐way ANOVA and Tukey tests were performed for *post hoc* analysis. *P < 0.05, **P < 0.01, ****P < 0.0001. (E) Colony formation assays were conducted. The TAK‐931 treatment duration was 10 days. Representative images are shown. Cells treated with 0.1% DMSO were used as the negative control.

Phosphorylation of MCM2 by cell division cycle 7 (CDC7) at the G_1_ phase to S phase initiates DNA synthesis [42]. CDC7 is a dumbbell former 4 protein (DBF4)‐dependent serine/threonine kinase and plays important roles in DNA replication [43]. CDC7 kinase activity is essential for MCM2‐MCM7 hexamer‐induced DNA replication. We hypothesized that indirect suppression of MCM2‐MCM7 hexamer function caused by CDC7 inhibition may decrease tumor cell progression (Fig. 8A). TAK‐931 (simurosertib) was used as a CDC7 inhibitor in this study.

In A549 and H1299 cells, TAK‐931 blocked the phosphorylation of MCM2 (Ser40) in a concentration‐dependent manner (Fig. 8B; Fig. S6). Moreover, cell cycle assays showed that cells treated with TAK‐931 exhibited late S to late G_2_/M arrest in a concentration‐ and time‐dependent manner (Fig. 8C; Fig. S7). This result suggested that TAK‐931 suppressed the functions of MCM2‐MCM7 and that DNA replication was delayed in A549 and H1299 cells.

Cytotoxicity was evaluated using XTT assays. After treatment with TAK‐931, cell viability decreased significantly when the concentration of TAK‐931 was greater than 100 nm (Fig. 8D). In 10‐day colony formation assays, TAK‐931 inhibited colony formation in a concentration‐dependent manner (Fig. 8E). We found that inhibition of CDC7 suppressed tumor cell progression. CDC7 inhibitors may be another treatment strategy for NSCLC based on these findings.

## Discussion

DNA replication is the most critical issue for the normal maintenance of cells, and its regulation is extremely strict. In cancer cells, however, DNA replication proceeds chaotically, which triggers abnormal cell proliferation. Aberrant expression of various genes involved in DNA replication has been reported in cancer cells [44, 45].

The hexameric protein complex formed by MCM proteins (MCM2–MCM7) is a key component of the prereplication complex and recruits other DNA replication‐associated proteins to form replication forks [42, 46]. Aberrant expression of MCM proteins has been reported in several types of cancers, for example, renal cell carcinoma, prostate cancer, and breast cancer, and these events promote genome instability and uncontrolled cell cycle progression [23, 47]. Several sets of cohort data in TCGA for LUAD revealed that all MCM family members were significantly upregulated in LUAD tissues. Moreover, *MCM4*, *MCM5*, and *MCM7* expression levels may be used as prognostic molecular markers for patients with LUAD. In the future, these molecules may have applications in predicting the efficacy of treatment regimens and in the selection of therapeutic drugs for patients with LUAD.

Minichromosome maintenance family members have been reported to be phosphorylated by various kinases and activated in human cells [44, 48]. Aberrant activation of the MCM family by excess phosphorylation causes disruption of DNA replication and the cell cycle and is involved in the development and progression of cancer cells [49, 50]. MCM7 forms a complex with MCM2, MCM4, and MCM6 and has been shown to regulate the helicase activity of the complex [51]. Aberrant expression of MCM7 has been reported in several types of cancers, and its expression leads to a poor prognosis in these patients [52, 53]. MCM7 phosphorylation (Y600) through the epidermal growth factor/Lyn kinase cascade enhances MCM complex assembly and cancer cell proliferation [49]. RACK1 is a highly conserved WD40 repeat scaffold protein that is involved in several cellular processes [50]. Overexpression of RACK1 promotes AKT‐dependent phosphorylation of MCM7 and is involved in DNA replication and proliferation in NSCLC cells [50].

CDC7, a serine/threonine kinase, regulates DNA replication, cell cycle, DNA repair, and gene expression by phosphorylating many intracellular target molecules [42, 54]. Notably, MCM is a key target of CDC7, and the CDC7/DBF4 complex selectively phosphorylates MCM2 (Ser40 and Ser53) and is involved in regulating the initiation of DNA replication [55, 56]. Therefore, CDC7 is a therapeutic target molecule for cancer therapy, and various CDC7 inhibitors have been developed [42]. TAK‐931 is a novel CDC7‐selective inhibitor that has been shown to exhibit significant antitumor effects both *in vitro* and *in vivo* [57]. Our data showed that treatment of LUAD cells with TAK‐931 markedly suppressed cell proliferation by targeting MCM2 phosphorylation. The development of therapeutic agents that target the CDC7/MCM2 cascade will contribute to improving the prognosis of patients with LUAD.

Based on the epigenomic perspective, we further investigated the cause of *MCM2* upregulation in LUAD cells. Numerous studies have clarified that noncoding RNAs are involved in the regulation of gene expression. In particular, miRNAs play pivotal roles as regulators of gene expression [24, 25]. Previous studies have shown that *miR‐186‐3p* is downregulated in cervical cancer tissues and cell lines and that the expression of this miRNA directly controls *MCM2* expression [58]. In colon cancer, *MCM2* expression is regulated by *miR‐195‐5p* and *miR‐497‐5p*, which are downregulated in cancer tissues and cultured cancer stem cells. Overexpression of *MCM2* is involved in the maintenance of stemness [59].

Our current findings showed that *miR‐139‐3p*, *miR‐378a‐5p*, and *miR‐2110* were downregulated in LUAD tissues and controlled the expression of *MCM2* in LUAD cells. Previous studies have demonstrated that *miR‐139‐3p* was downregulated in lung squamous cell carcinoma tissues and that checkpoint kinase 1 (*CHEK1*) could be targeted by *miR‐139‐3p* [60]. Notably, CHEK1 is closely involved in various biological processes, for example, cell cycle arrest, DNA repair, and cell death [61, 62].

The Cancer Genome Atlas analysis revealed that aberrant expression of *MCM* genes (*MCM2*–*MCM7*) has a negative impact on prognosis with LUAD patients. In this study, we showed candidate miRNAs that negatively regulate *MCM* genes. For example, *miR‐144‐3p*, *miR‐2110*, *miR‐30c‐2‐3p*, etc. may regulate multiple *MCMs* from our data (Table 4 and Tables [Link], [Link]), and some of them are also downregulated in TCGA‐LUAD cohort dataset. Functional analyses of these miRNAs will be a future challenge. Elucidation of the molecular networks regulated by tumor‐suppressive miRNAs will reveal the novel oncogenic pathways underlying the malignant transformation of LUAD.

## Conclusions

Analysis of TGCA‐LUAD datasets and immunostaining revealed that all MCM family members (MCM2–MCM7) were overexpressed in cancer tissues. Notably, high expression of *MCM4*, *MCM5*, and *MCM7* significantly predicted 5‐year survival rates in patients with LUAD. Functional assays showed that knockdown of each *MCM* gene significantly suppressed the proliferation of LUAD cells. The CDC7‐selective inhibitor TAK‐931 attenuated the proliferation of LUAD cells by suppressing MCM2 phosphorylation. Moreover, *MCM2* expression was controlled by *miR‐139‐3p*, *miR‐378a‐5p*, and *miR‐2110*. MCM family members have DNA helicase activity that is essential for the replication of genomic DNA and are closely involved in the molecular pathogenesis of LUAD. In particular, regulation of MCM2 phosphorylation may be a promising target when developing novel treatments for this disease.

## Conflict of interest

The authors declare no conflict of interest.

### Peer review

The peer review history for this article is available at https://www.webofscience.com/api/gateway/wos/peer‐review/10.1002/2211‐5463.13681.

## Author contributions

KT, KM, HI, and NS conceived the study and designed the experiments. KT, TS, and NS wrote the manuscript. KT, YT, and SM performed the experiments. KT, YT, NK, SA, and YH analyzed the data. All authors read and approved the manuscript.

## Supporting information


**Fig. S1.** Selection of miRNAs regulating *MCM3‐MCM7* in LUAD cells.

**Fig. S2.** mRNA and protein expression of MCM2, MCM3, MCM4, MCM5, MCM6, and MCM7 after transfection with siRNAs into NSCLC cells.

**Fig. S3.** Full‐size images of the western blots shown in Fig. S2.

**Fig. S4.** Histogram of cell cycle assays after transfection of A549 cells with siRNAs targeting *MCM2*, *MCM3*, *MCM4*, *MCM5*, *MCM6*, and *MCM7*.

**Fig. S5.** Histogram of cell cycle assays after transfection of H1299 cells with siRNAs targeting *MCM2*, *MCM3*, *MCM4*, *MCM5*, *MCM6*, and *MCM7*.

**Fig. S6.** Full‐size images of the western blots shown in Fig. 8.

**Fig. S7.** Effects of CDC7 inhibition on MCM2 phosphorylation and late S to early G_2_/M cell cycle arrest in NSCLC cell lines.

**Table S1.** Reagents used in this study.

**Table S2.** Characteristics of the patients used for IHC.

**Table S3.** Candidate tumor‐suppressive miRNAs binding to *MCM3*.

**Table S4.** Candidate tumor‐suppressive miRNAs binding to *MCM4*.

**Table S5.** Candidate tumor‐suppressive miRNAs binding to *MCM5*.

**Table S6.** Candidate tumor‐suppressive miRNAs binding to *MCM6*.

**Table S7.** Candidate tumor‐suppressive miRNAs binding to *MCM7*.

## Data Availability

The data that support the findings of this study are openly available in NCBI's Gene Expression Omnibus and are accessible through https://www.ncbi.nlm.nih.gov/geo/query/acc.cgi?acc=GSE19188, GEO Series accession number GSE19188; https://www.ncbi.nlm.nih.gov/geo/query/acc.cgi?acc=GSE230229, GEO Series accession number GSE230229.
