# Supplementary figures and images for: Minichromosome maintenance proteins in lung adenocarcinoma: Clinical significance and therapeutic targets
Source: FEBS Open Bio. 2023 Aug 7;13(9):1737–55. doi: 10.1002/2211-5463.13681 (PMC10476565; doi:10.1002/2211-5463.13681)

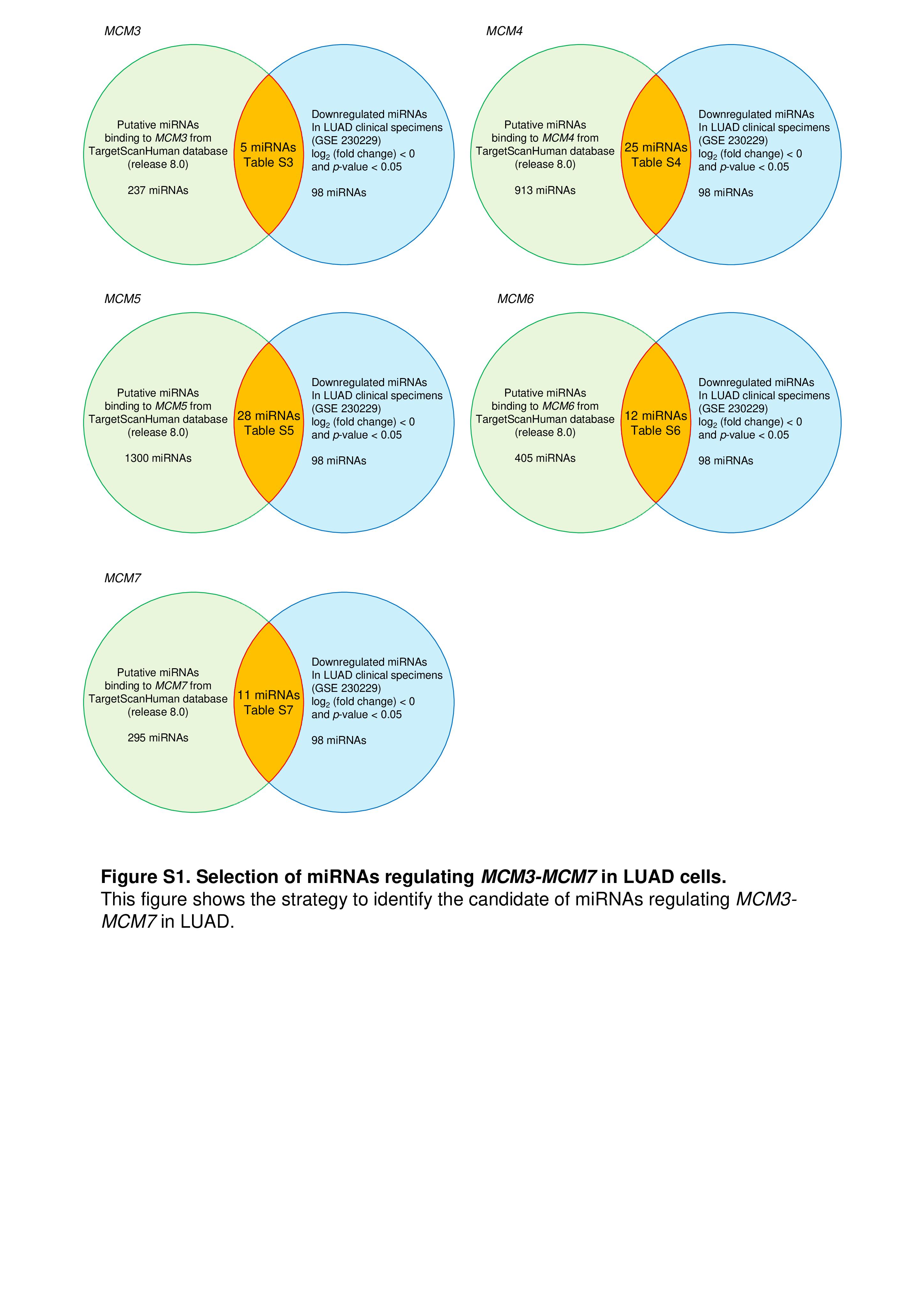

Supplement: Supplementary file 1 — Fig. S1. Selection of miRNAs regulating MCM3‐MCM7 in LUAD cells. [file FEB4-13-1737-s014.jpg]

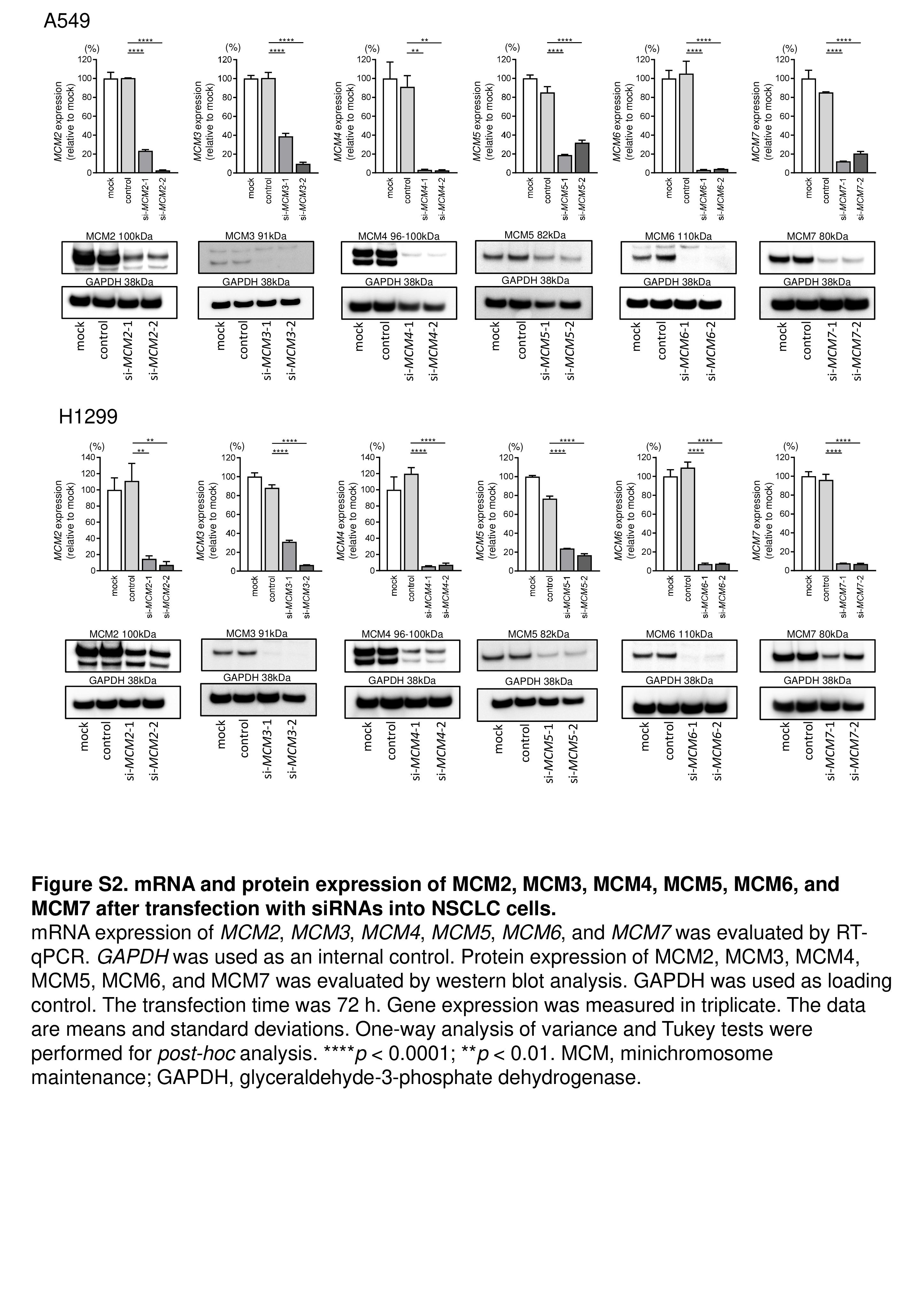

Supplement: Supplementary file 2 — Fig. S2. mRNA and protein expression of MCM2, MCM3, MCM4, MCM5, MCM6, and MCM7 after transfection with siRNAs into NSCLC cells. [file FEB4-13-1737-s007.jpg]

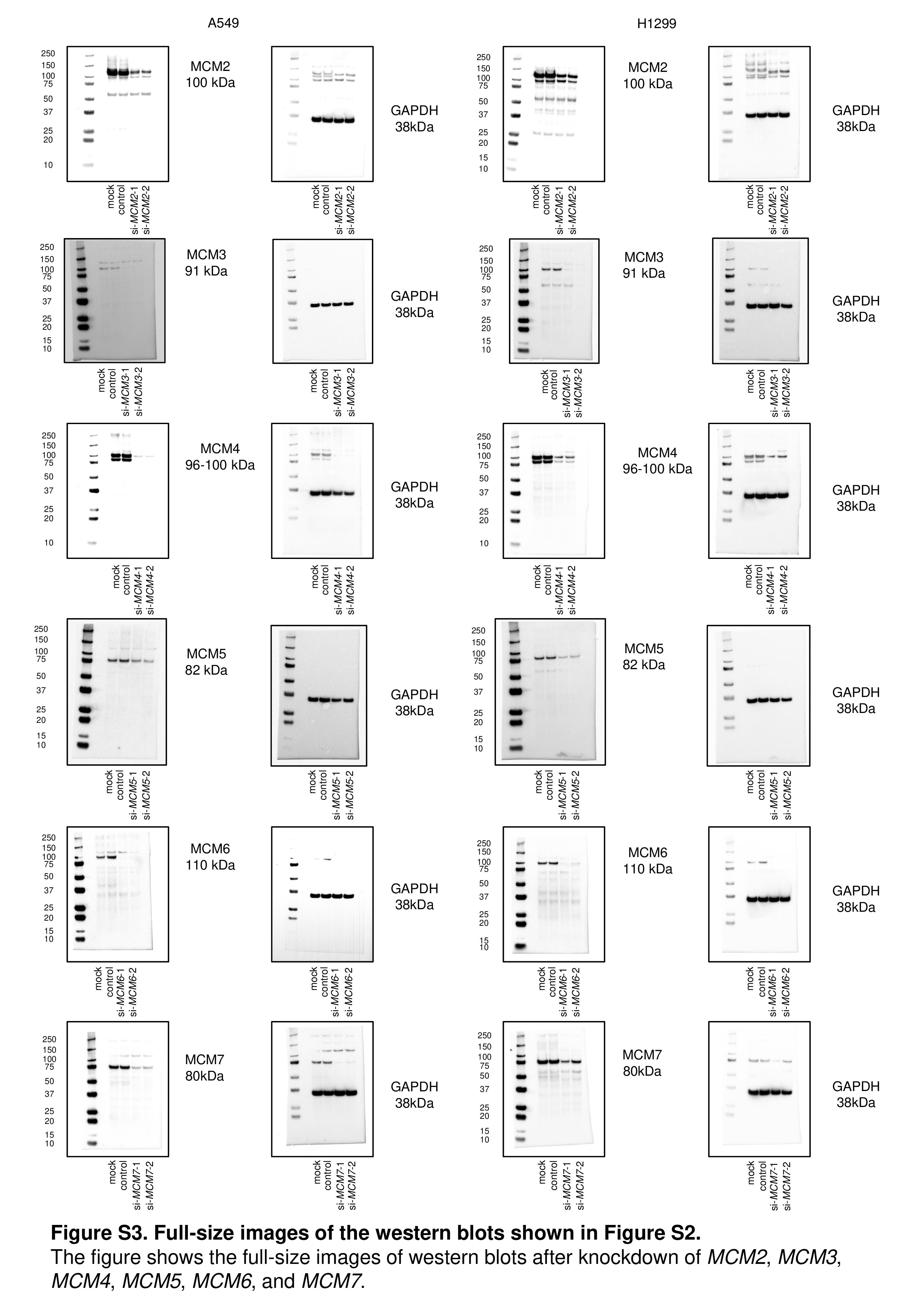

Supplement: Supplementary file 3 — Fig. S3. Full‐size images of the western blots shown in Fig. S2. [file FEB4-13-1737-s009.jpg]

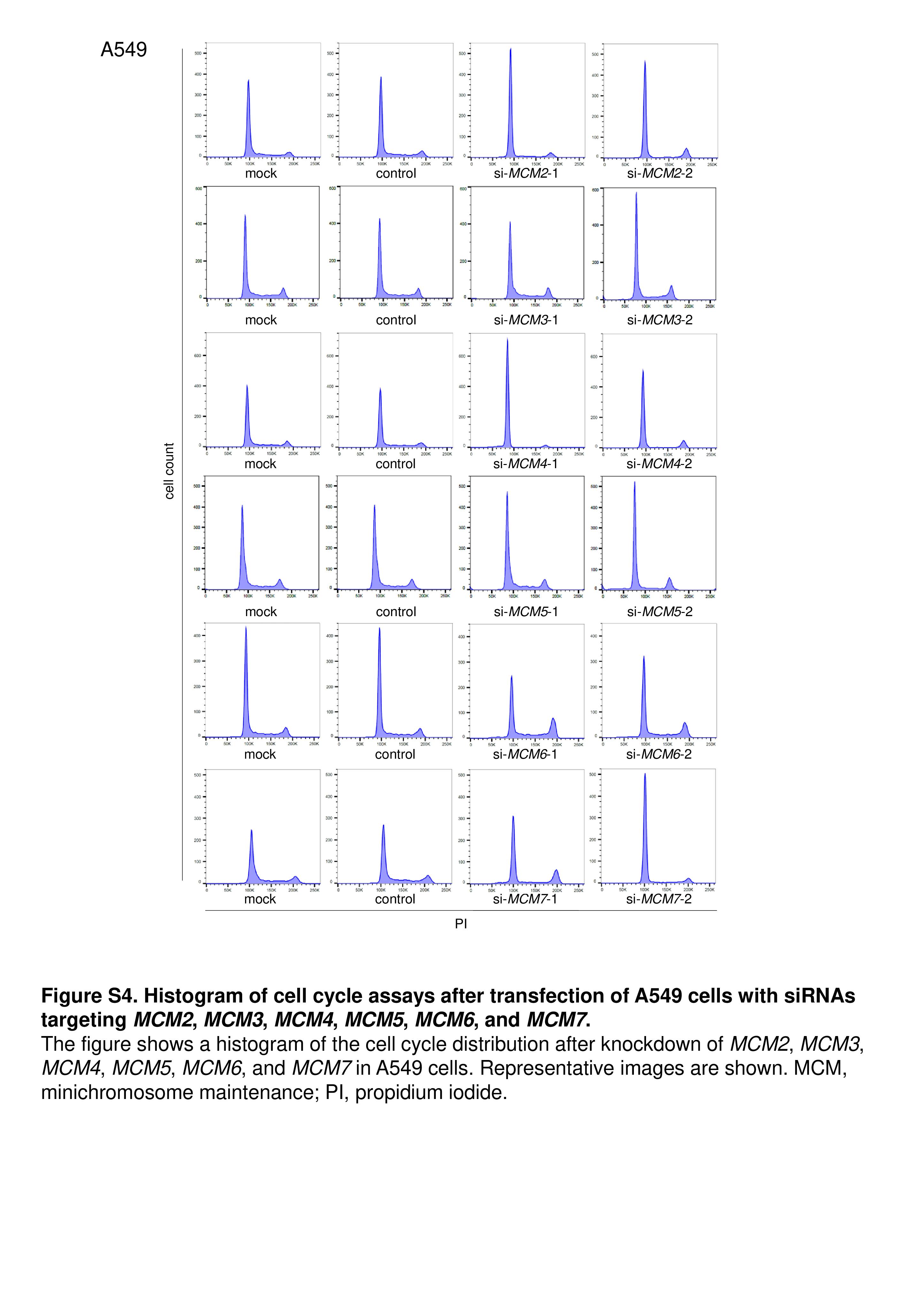

Supplement: Supplementary file 4 — Fig. S4. Histogram of cell cycle assays after transfection of A549 cells with siRNAs targeting MCM2, MCM3, MCM4, MCM5, MCM6, and MCM7. [file FEB4-13-1737-s005.jpg]

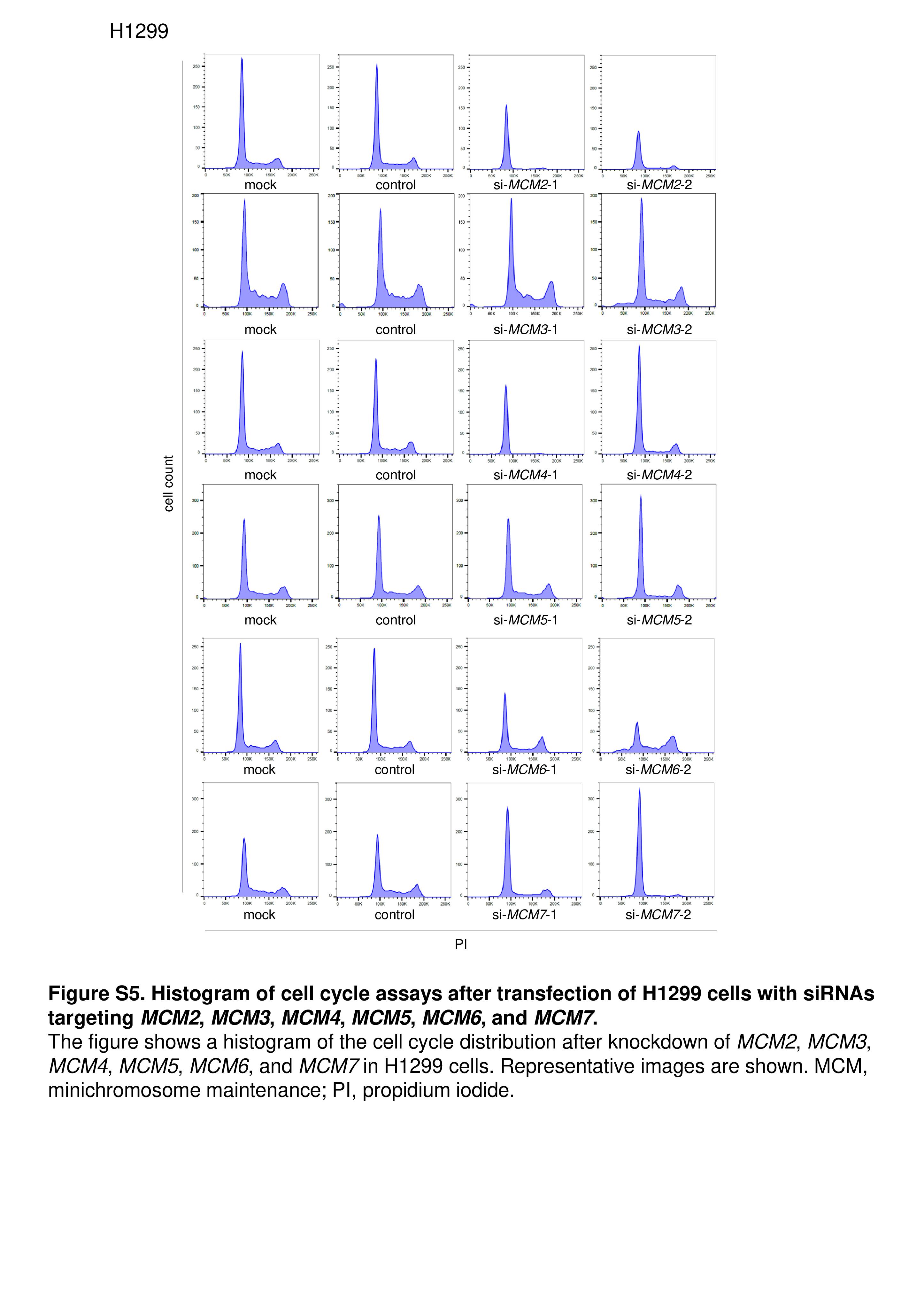

Supplement: Supplementary file 5 — Fig. S5. Histogram of cell cycle assays after transfection of H1299 cells with siRNAs targeting MCM2, MCM3, MCM4, MCM5, MCM6, and MCM7. [file FEB4-13-1737-s010.jpg]

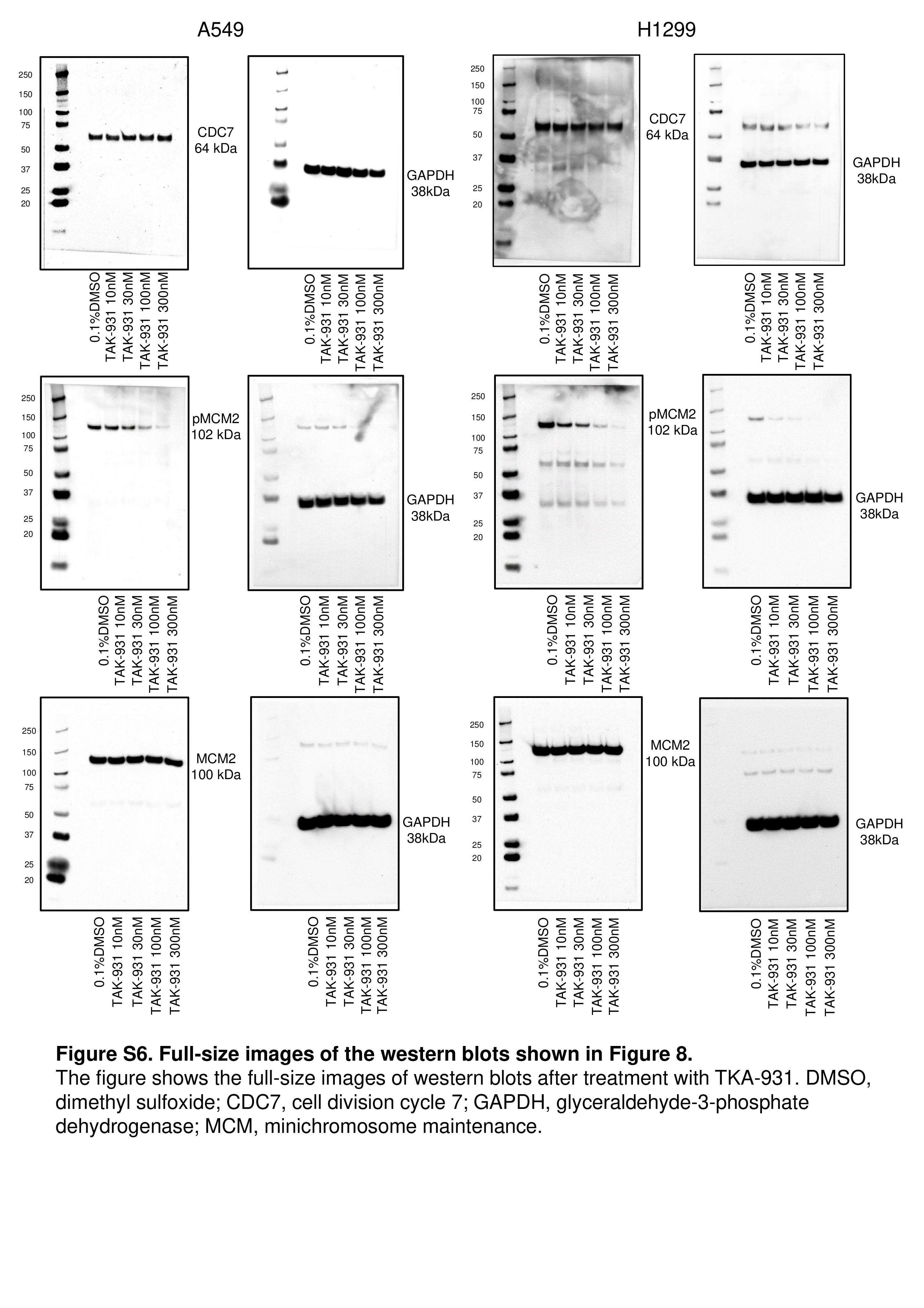

Supplement: Supplementary file 6 — Fig. S6. Full‐size images of the western blots shown in Fig. 8. [file FEB4-13-1737-s012.jpg]

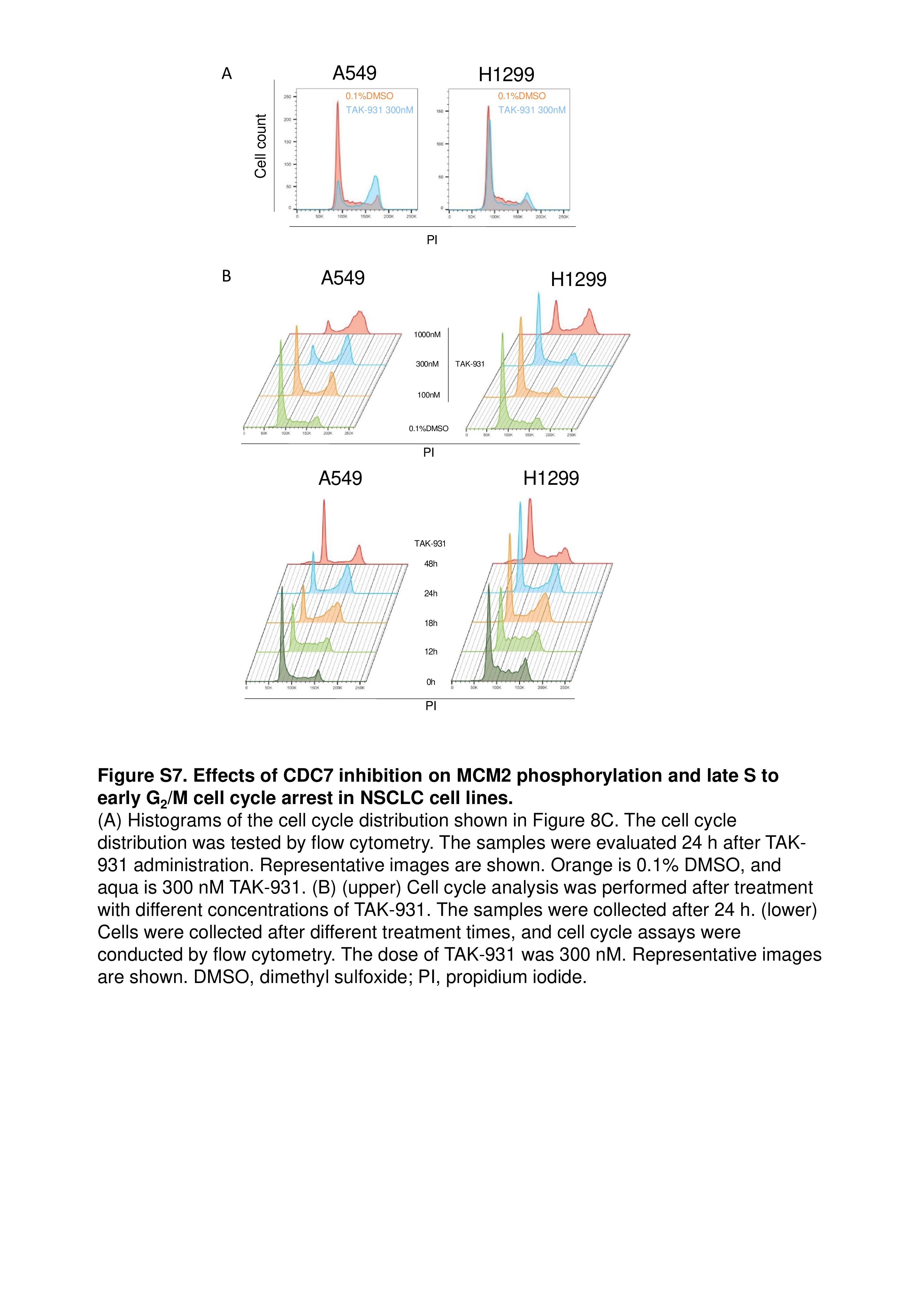

Supplement: Supplementary file 7 — Fig. S7. Effects of CDC7 inhibition on MCM2 phosphorylation and late S to early G2/M cell cycle arrest in NSCLC cell lines. [file FEB4-13-1737-s004.jpg]
