# Supplementary material for: Minichromosome maintenance proteins in lung adenocarcinoma: Clinical significance and therapeutic targets
Source: FEBS Open Bio. 2023 Aug 7;13(9):1737–55. doi: 10.1002/2211-5463.13681 (PMC10476565; doi:10.1002/2211-5463.13681)
Supplement: Supplementary file 8 — Table S1. Reagents used in this study. [file FEB4-13-1737-s008.pdf]

**Table S1. Reagents used in this study.**

| Probe | | Assay ID | Company |
|---|---|---|---|
| *MCM2* | | Hs01091564_m1 | Applied Biosystem (Foster City, CA, USA) |
| *MCM3* | | Hs00172459_m1 | Applied Biosystem |
| *MCM4* | | Hs00908398_m1 | Applied Biosystem |
| *MCM5* | | Hs01052148_m1 | Applied Biosystem |
| *MCM6* | | Hs00962418_m1 | Applied Biosystem |
| *MCM7* | | Hs00428518_m1 | Applied Biosystem |
| *GAPDH* | | Hs99999905_m1 | Applied Biosystem |
| **siRNA** | **Concentration** | **Catalog number<br>primer name or sequence (5' to 3')** | **Company** |
| si-*MCM2* | 10nM | Cat#10620312<br>HSS106390, HSS181044 | Invitrogen (Carlsbad, CA, USA) |
| si-*MCM3* | 10nM | Cat#10620312<br>HSS181045, GAACCUACCGUUGCCUUCCUGGAAA | Invitrogen |
| si-*MCM4* | 10nM | Cat#10620312<br>HSS106396, HSS106398 | Invitrogen |
| si-*MCM5* | 10nM | Cat#10620312<br>HSS181047, GAGUGCUCCGGUUUCUUCAUCUGUU | Invitrogen |
| si-*MCM6* | 10nM | Cat#10620312<br>HSS106403, HSS106404 | Invitrogen |
| si-*MCM7* | 10nM | Cat#10620312<br>HSS106405, HSS181048 | Invitrogen |
| **Antibody** | **Dilution** | **Catalog number** | **Company** |
| MCM2 | WB 1:1000<br>IHC 1:1000 | ab4461 | Abcam (Cambridge, UK) |
| pMCM2 | WB 1:1000 | ab133243 | Abcam |
| MCM3 | WB 1:1000<br>IHC 1:100 | ab128923 | Abcam |
| MCM4 | WB 1:2000<br>IHC 1:1000 | ab4459 | Abcam |
| MCM5 | WB 1:1000<br>IHC 1:50 | ab75975 | Abcam |
| MCM6 | WB 1:2000<br>IHC 1:100 | ab4458 | Abcam |
| MCM7 | WB 1:1000<br>IHC 1:1000 | ab2360 | Abcam |
| CDC7 | WB 1:500 | ab229187 | Abcam |
| GAPDH | WB 1:20000 | MAB374 | EMD Millipore (Billerica, MA, USA) |
| **Inhibitor** | | **catalog number** | **company** |
| TAK-931 (simurosertib) | | HY-100888 | MedChemExpress (Monmouth Junction, NJ, USA) |
| **miRNA** | **Concentration** | **Catalog number / Assay ID** | **company** |
| *miR-139-3p* | 10nM | AM17100 / PM25489 | Invitrogen |
| *miR-378a-5p* | 10nM | AM17100 / PM10049 | Invitrogen |
| *miR-2110* | 10nM | AM17100 / PM14979 | Invitrogen |
| anti-miR Negative Control #1 | 10nM | AM17100 / - | Invitrogen |
