# Supplementary material for: Minichromosome maintenance proteins in lung adenocarcinoma: Clinical significance and therapeutic targets
Source: FEBS Open Bio. 2023 Aug 7;13(9):1737–55. doi: 10.1002/2211-5463.13681 (PMC10476565; doi:10.1002/2211-5463.13681)
Supplement: Supplementary file 9 — Table S2. Characteristics of the patients used for IHC. [file FEB4-13-1737-s006.pdf]

**Table S2: Characteristics of the patients used for IHC.**

| No. | Age | Sex | T | N | M | Pathological stage | Type |
|---|---|---|---|---|---|---|---|
| 1 | 64 | F | 2 | 1 | 0 | IIA | LUAD |
| 2 | 54 | F | 2 | 1 | 0 | IIA | LUAD |
| 3 | 50 | F | 2 | 0 | 0 | IB | LUAD |
| 4 | 70 | M | 1 | 0 | 0 | IA | LUAD |
| 5 | 65 | F | 2 | 2 | 0 | IIIA | LUAD |
| 6 | 55 | M | 2 | 0 | 0 | IB | LUAD |
| 7 | 56 | F | 1b | 0 | 0 | IA | LUAD |
| 8 | 77 | M | 3 | 0 | 0 | IIB | LUAD |
| 9 | 62 | M | 2a | 0 | 0 | IB | LUAD |
| 10 | 71 | F | 2 | 1 | 0 | IIA | LUAD |
| 11 | 35 | F | 2 | 1 | 0 | IIA | LUAD |
| 12 | 59 | M | 4 | 3 | 0 | IIIB | LUAD |
| 13 | 43 | F | 2 | 2 | 0 | IIIA | LUAD |
| 14 | 58 | M | 1 | 0 | 0 | IA | LUAD |
| 15 | 43 | M | 2a | 0 | 0 | IB | LUAD |
| 16 | 49 | F | 2 | 1 | 0 | IIA | LUAD |
| 17 | 75 | M | 2 | 0 | 0 | IB | LUAD |
| 18 | 65 | M | 2 | 2 | 0 | IIIA | LUAD |
| 19 | 53 | M | 2 | 0 | 0 | IB | LUAD |
| 20 | 67 | M | 2 | 0 | 0 | IB | LUAD |
| 21 | 57 | M | 2 | 0 | 0 | IB | LUAD |
| 22 | 82 | M | 4 | 1 | 0 | IIIA | LUAD |
| 23 | 52 | F | 3 | 1 | 0 | IIIA | LUAD |
| 24 | 60 | F | 2 | 0 | 0 | IB | LUAD |
| 25 | 54 | M | 3 | 1 | 0 | IIIA | LUAD |
| 26 | 47 | M | 2 | 0 | 0 | IB | LUAD |
| 27 | 57 | F | 2 | 0 | 0 | IB | LUAD |
| 28 | 56 | F | 2 | 0 | 0 | IB | LUAD |
| 29 | 56 | F | 2 | 1 | 0 | IIA | LUAD |
| 30 | 67 | M | 2 | 0 | 0 | IB | LUAD |
| 31 | 50 | F | 2 | 0 | 0 | IB | LUAD |
| 32 | 48 | F | 2 | 0 | 0 | IB | LUAD |
| 33 | 67 | M | 2 | 1 | 0 | IIA | LUAD |
| 34 | 75 | F | 2 | 1 | 0 | IIA | LUAD |
| 71 | 40 | F | - | - | - | - | normal lung tissue |
| 72 | 23 | M | - | - | - | - | normal lung tissue |
| 73 | 33 | M | - | - | - | - | normal lung tissue |
| 74 | 30 | M | - | - | - | - | normal lung tissue |
| 75 | 16 | F | - | - | - | - | normal lung tissue |
| 76 | 30 | M | - | - | - | - | normal lung tissue |
| 77 | 21 | F | - | - | - | - | normal lung tissue |
| 78 | 2 | F | - | - | - | - | normal lung tissue |
| 79 | 24 | M | - | - | - | - | normal lung tissue |
| 80 | 48 | M | - | - | - | - | normal lung tissue |

IHC: immunohistochemistry.

LUAD: lung adenocarcinoma.
