# Supplementary material for: Minichromosome maintenance proteins in lung adenocarcinoma: Clinical significance and therapeutic targets
Source: FEBS Open Bio. 2023 Aug 7;13(9):1737–55. doi: 10.1002/2211-5463.13681 (PMC10476565; doi:10.1002/2211-5463.13681)
Supplement: Supplementary file 10 — Table S3. Candidate tumor‐suppressive miRNAs binding to MCM3. [file FEB4-13-1737-s013.pdf]

Table S3. Candidate tumor-suppressive miRNAs binding to *MCM3*.

MicroRNA	miRBase accession No.	Log_2 fold change GSE230229	Normalized read count GSE230229		FDR GSE230229	*p* value GSE230229
			LUAD tissues	Normal lung tissues		
hsa-miR-1208	MIMAT0005873	-2.95	0.00	2.95	0.002	<0.001
hsa-miR-516b-5p	MIMAT0002859	-2.50	1.12	3.62	0.100	0.022
hsa-miR-373-3p	MIMAT0000726	-2.09	0.00	2.09	0.152	0.038
hsa-miR-4516	MIMAT0019053	-1.38	3.56	4.94	0.164	0.042
hsa-miR-3613-3p	MIMAT0017991	-1.25	5.14	6.40	0.016	0.003

LUAD: lung adenocarcinoma

FDR: false discovery rate
