# Supplementary material for: Minichromosome maintenance proteins in lung adenocarcinoma: Clinical significance and therapeutic targets
Source: FEBS Open Bio. 2023 Aug 7;13(9):1737–55. doi: 10.1002/2211-5463.13681 (PMC10476565; doi:10.1002/2211-5463.13681)
Supplement: Supplementary file 11 — Table S4. Candidate tumor‐suppressive miRNAs binding to MCM4. [file FEB4-13-1737-s003.pdf]

Table S4. Candidate tumor-suppressive miRNAs binding to *MCM4*.

MicroRNA	miRBase accession No.	Log_2 fold change GSE230229	Normalized read count GSE230229		FDR GSE230229	*p* value GSE230229
			LUAD tissues	Normal lung tissues		
hsa-miR-4795-3p	MIMAT0019969	-2.95	0.00	2.95	<0.001	<0.001
hsa-miR-1208	MIMAT0005873	-2.95	0.00	2.95	0.002	<0.001
hsa-miR-603	MIMAT0003271	-2.90	0.00	2.90	<0.001	<0.001
hsa-miR-1258	MIMAT0005909	-2.31	1.36	3.67	0.158	0.040
hsa-miR-4529-5p	MIMAT0019236	-2.22	1.15	3.37	0.120	0.028
hsa-miR-6768-5p	MIMAT0027436	-2.13	4.12	6.25	0.076	0.016
hsa-miR-3622a-5p	MIMAT0018003	-2.12	0.95	3.07	0.141	0.034
hsa-miR-373-3p	MIMAT0000726	-2.09	0.00	2.09	0.152	0.038
hsa-miR-144-3p	MIMAT0000436	-2.06	10.85	12.91	0.107	0.024
hsa-miR-584-5p	MIMAT0003249	-1.79	5.73	7.52	0.008	0.001
hsa-miR-4524a-3p	MIMAT0019063	-1.59	4.16	5.76	0.064	0.013
hsa-miR-139-5p	MIMAT0000250	-1.59	7.51	9.11	0.078	0.016
hsa-miR-4705	MIMAT0019805	-1.40	5.72	7.11	0.112	0.025
hsa-miR-4516	MIMAT0019053	-1.38	3.56	4.94	0.164	0.042
hsa-miR-126-5p	MIMAT0000444	-1.31	16.10	17.41	0.142	0.034
hsa-miR-143-3p	MIMAT0000435	-1.27	17.97	19.24	0.084	0.018
hsa-miR-3613-3p	MIMAT0017991	-1.25	5.14	6.40	0.016	0.003
hsa-miR-145-3p	MIMAT0004601	-1.24	11.26	12.50	0.157	0.039
hsa-miR-143-5p	MIMAT0004599	-1.14	11.48	12.62	0.172	0.044
hsa-miR-150-3p	MIMAT0004610	-1.10	3.95	5.05	0.087	0.018
hsa-miR-548h-3p	MIMAT0022723	-1.01	8.04	9.04	0.180	0.047
hsa-miR-1273h-3p	MIMAT0030416	-0.96	3.93	4.89	0.144	0.035
hsa-miR-6872-3p	MIMAT0027645	-0.87	7.07	7.93	0.022	0.004
hsa-miR-203b-5p	MIMAT0019813	-0.82	8.79	9.61	0.148	0.036
hsa-miR-378a-5p	MIMAT0000731	-0.80	8.58	9.38	0.010	0.002

LUAD: lung adenocarcinoma

FDR: false discovery rate
