# Supplementary material for: Minichromosome maintenance proteins in lung adenocarcinoma: Clinical significance and therapeutic targets
Source: FEBS Open Bio. 2023 Aug 7;13(9):1737–55. doi: 10.1002/2211-5463.13681 (PMC10476565; doi:10.1002/2211-5463.13681)
Supplement: Supplementary file 12 — Table S5. Candidate tumor‐suppressive miRNAs binding to MCM5. [file FEB4-13-1737-s011.pdf]

Table S5. Candidate tumor-suppressive miRNAs binding to *MCM5*.

MicroRNA	miRBase accession No.	Log_2 fold change GSE230229	Normalized read count GSE230229		FDR GSE230229	*p* value GSE230229
			LUAD tissues	Normal lung tissues		
hsa-miR-551b-5p	MIMAT0004794	-3.39	1.03	4.42	0.022	0.004
hsa-miR-4703-3p	MIMAT0019802	-3.15	0.00	3.15	<0.001	<0.001
hsa-miR-4795-3p	MIMAT0019969	-2.95	0.00	2.95	<0.001	<0.001
hsa-miR-1208	MIMAT0005873	-2.95	0.00	2.95	0.002	<0.001
hsa-miR-603	MIMAT0003271	-2.90	0.00	2.90	<0.001	<0.001
hsa-miR-486-3p	MIMAT0004762	-2.86	5.44	8.30	0.016	0.003
hsa-miR-34b-5p	MIMAT0000685	-2.73	9.78	12.52	0.175	0.046
hsa-miR-4532	MIMAT0019071	-2.70	8.57	11.26	0.106	0.024
hsa-miR-6813-3p	MIMAT0027527	-2.65	0.00	2.65	0.007	0.001
hsa-miR-940	MIMAT0004983	-2.63	1.30	3.92	0.127	0.030
hsa-miR-371b-3p	MIMAT0019893	-2.50	1.49	3.99	0.178	0.047
hsa-miR-4483	MIMAT0019017	-2.36	3.36	5.72	0.141	0.034
hsa-miR-4529-5p	MIMAT0019236	-2.22	1.15	3.37	0.120	0.028
hsa-miR-144-3p	MIMAT0000436	-2.06	10.85	12.91	0.107	0.024
hsa-miR-138-5p	MIMAT0000430	-1.63	9.35	10.98	0.104	0.023
hsa-miR-1-3p	MIMAT0000416	-1.60	11.03	12.63	0.184	0.049
hsa-miR-139-5p	MIMAT0000250	-1.59	7.51	9.11	0.078	0.016
hsa-miR-145-5p	MIMAT0000437	-1.44	14.95	16.39	0.159	0.040
hsa-miR-451b	MIMAT0019840	-1.39	4.03	5.41	0.109	0.025
hsa-miR-4516	MIMAT0019053	-1.38	3.56	4.94	0.164	0.042
hsa-miR-126-5p	MIMAT0000444	-1.31	16.10	17.41	0.142	0.034
hsa-miR-887-3p	MIMAT0004951	-1.31	6.25	7.56	0.006	0.001
hsa-miR-143-3p	MIMAT0000435	-1.27	17.97	19.24	0.084	0.018
hsa-miR-548ao-5p	MIMAT0021029	-1.12	4.55	5.67	0.003	0.000
hsa-miR-484	MIMAT0002174	-1.04	7.73	8.77	0.055	0.011
hsa-miR-548h-3p	MIMAT0022723	-1.01	8.04	9.04	0.180	0.047
hsa-miR-203b-5p	MIMAT0019813	-0.82	8.79	9.61	0.148	0.036
hsa-miR-2110	MIMAT0010133	-0.69	6.91	7.59	0.141	0.034

LUAD: lung adenocarcinoma

FDR: false discovery rate
