# Supplementary material for: Minichromosome maintenance proteins in lung adenocarcinoma: Clinical significance and therapeutic targets
Source: FEBS Open Bio. 2023 Aug 7;13(9):1737–55. doi: 10.1002/2211-5463.13681 (PMC10476565; doi:10.1002/2211-5463.13681)
Supplement: Supplementary file 13 — Table S6. Candidate tumor‐suppressive miRNAs binding to MCM6. [file FEB4-13-1737-s002.pdf]

Table S6. Candidate tumor-suppressive miRNAs binding to *MCM6*.

MicroRNA	miRBase accession No.	Log_2 fold change GSE230229	Normalized read count GSE230229		FDR GSE230229	*p* value GSE230229
			LUAD tissues	Normal lung tissues		
hsa-miR-551b-5p	MIMAT0004794	-3.39	1.03	4.42	0.022	0.004
hsa-miR-218-1-3p	MIMAT0004565	-3.07	1.05	4.12	0.061	0.012
hsa-miR-6813-3p	MIMAT0027527	-2.65	0.00	2.65	0.007	0.001
hsa-miR-6768-5p	MIMAT0027436	-2.13	4.12	6.25	0.076	0.016
hsa-miR-373-3p	MIMAT0000726	-2.09	0.00	2.09	0.152	0.038
hsa-miR-144-3p	MIMAT0000436	-2.06	10.85	12.91	0.107	0.024
hsa-miR-30c-2-3p	MIMAT0004550	-1.92	8.27	10.19	0.119	0.027
hsa-miR-1-5p	MIMAT0031892	-1.62	3.76	5.37	0.047	0.009
hsa-miR-145-3p	MIMAT0004601	-1.24	11.26	12.50	0.157	0.039
hsa-miR-548ao-5p	MIMAT0021029	-1.12	4.55	5.67	0.003	<0.001
hsa-miR-484	MIMAT0002174	-1.04	7.73	8.77	0.055	0.011
hsa-miR-2110	MIMAT0010133	-0.69	6.91	7.59	0.141	0.034

LUAD: lung adenocarcinoma

FDR: false discovery rate
