# Supplementary material for: Minichromosome maintenance proteins in lung adenocarcinoma: Clinical significance and therapeutic targets
Source: FEBS Open Bio. 2023 Aug 7;13(9):1737–55. doi: 10.1002/2211-5463.13681 (PMC10476565; doi:10.1002/2211-5463.13681)
Supplement: Supplementary file 14 — Table S7. Candidate tumor‐suppressive miRNAs binding to MCM7. [file FEB4-13-1737-s001.pdf]

Table S7. Candidate tumor-suppressive miRNAs binding to *MCM7*.

MicroRNA	miRBase accession No.	Log_2 fold change GSE230229	Normalized read count GSE230229		FDR GSE230229	*p* value GSE230229
			LUAD tissues	Normal lung tissues		
hsa-miR-519a-3p	MIMAT0002869	-2.90	0.00	2.90	0.012	0.002
hsa-miR-486-3p	MIMAT0004762	-2.86	5.44	8.30	0.016	0.003
hsa-miR-34b-5p	MIMAT0000685	-2.73	9.78	12.52	0.175	0.046
hsa-miR-6768-5p	MIMAT0027436	-2.13	4.12	6.25	0.076	0.016
hsa-miR-373-3p	MIMAT0000726	-2.09	0.00	2.09	0.152	0.038
hsa-miR-30c-2-3p	MIMAT0004550	-1.92	8.27	10.19	0.119	0.027
hsa-miR-218-5p	MIMAT0000275	-1.65	12.92	14.57	0.103	0.023
hsa-miR-1-5p	MIMAT0031892	-1.62	3.76	5.37	0.047	0.009
hsa-miR-3613-3p	MIMAT0017991	-1.25	5.14	6.40	0.016	0.003
hsa-miR-548h-3p	MIMAT0022723	-1.01	8.04	9.04	0.180	0.047
hsa-miR-203b-5p	MIMAT0019813	-0.82	8.79	9.61	0.148	0.036

LUAD: lung adenocarcinoma

FDR: false discovery rate
